# Central Autonomic Dysfunction Delays Recovery of Fingolimod Induced Heart Rate Slowing

**DOI:** 10.1371/journal.pone.0132139

**Published:** 2015-07-06

**Authors:** Max J. Hilz, Tassanai Intravooth, Sebastian Moeller, Ruihao Wang, De-Hyung Lee, Julia Koehn, Ralf A. Linker

**Affiliations:** Department of Neurology, University of Erlangen-Nuremberg, Erlangen, Germany; Kurume University School of Medicine, JAPAN

## Abstract

**Background:**

In multiple sclerosis (MS) patients, Fingolimod may induce prolonged heart-rate slowing which might be caused by MS-related central autonomic lesions.

**Objectives:**

To evaluate whether MS-patients with prolonged heart-rate slowing (> six hours) upon Fingolimod show cardiovascular-autonomic dysfunction before Fingolimod-initiation.

**Methods:**

Before Fingolimod-initiation, we recorded electrocardiographic RR-intervals (RRIs) and blood-pressure (BP) at rest, upon standing-up, during metronomic deep-breathing, Valsalva-maneuver, and “sustained-handgrip-exercise” in 21 patients with relapsing-remitting MS, and 20 healthy persons. We calculated sympathetic and parasympathetic cardiovascular parameters, including low- (LF) and high-frequency (HF) powers of RRI- and BP-oscillations, RRI-RMSSDs, RRI- and BP-changes during handgrip-exercise, parasympathetic heart-rate-slowing in relation to BP-overshoot after Valsalva-strain-release. We compared values of healthy persons and patients with and without prolonged heart-rate slowing after Fingolimod-initiation (ANOVA; significance: p<0.05).

**Results:**

Upon Fingolimod-initiation, 7/21 patients had prolonged HR-slowing. Before Fingolimod, these patients had higher resting BP and higher BP increase during handgrip-exercise than had the other participants (p<0.05). They did not reduce parasympathetic HR-parameters upon standing-up. After Valsalva-strain-release, their parasympathetic HR-slowing in response to BP-overshoot was four times higher than in the other participants (p<0.05).

**Conclusions:**

The autonomic cardiovascular dysfunction in MS-patients with delayed HR-re-acceleration upon Fingolimod-initiation suggests that MS-related central autonomic lesions compromise HR-re-acceleration upon Fingolimod.

**Trial Registration:**

German Clinical Trial Register DRKS00004548 http://drks-neu.uniklinik-freiburg.de/drks_web/setLocale_EN.do

## Introduction

Fingolimod is an oral immunotherapy approved for treating patients with relapsing-remitting multiple sclerosis (MS) [1].

Fingolimod causes heart rate (HR) slowing even in healthy persons [2–5] and may cause prolonged or more prominent HR slowing in some MS patients [6]. In phase III trials [6], Fingolimod-initiation even caused bradycardia in 0.5–2.4 percent of patients, and cardiovascular serious adverse events in 0.9 percent, including atrioventricular blocks in 0.4 percent of patients [1,6,7]. HR-slowing upon Fingolimod-initiation very likely results from activation of G-protein-gated potassium channel I on atrial myocytes, due to structural similarity of Fingolimod to the Sphingosine 1 phosphate (SP1) opening these potassium channels [8,9]. Sodium efflux from myocytes and cell membrane hyperpolarization result in slowed depolarization and HR-slowing [10].

In healthy persons [2–5] and most MS patients [6,7,9], Fingolimod-induced HR-slowing is rather quickly counter-regulated and HR returns to baseline values within 4 to 6 hours [2,3,5]. Therefore, the European Medicines Agency recommends HR monitoring upon Fingolimod-initiation for at least six hours or if HR is at the lowest value 6 hours after the first dose, extended monitoring for at least 2 more hours and until HR increases again (http://www.ema.europa.eu/ema).

It is unknown why some MS patients have prolonged or more severe HR-slowing, or even develop bradycardia or atrioventricular blocks. It seems unlikely that this risk is primarily due to cardiac abnormalities such as individual variations in the sensitivity of myocardial potassium channels towards Fingolimod. Recently, Rossi et al. showed in 55 MS patients direct correlations between the nadir of HR slowing upon the first Fingolimod-intake and indices of parasympathetic activity assessed prior to Fingolimod-intake, such as the extent of HR slowing after a Valsalva maneuver or the extent of HR slowing and acceleration during deep metronomic breathing [11]. Conversely, the authors found indirect correlations between sympathetic cardiac modulation prior to Fingolimod-intake and increased electrocardiographic PR-intervals upon Fingolimod-intake indicating prolonged atrioventricular conduction. [12] The findings suggest that MS patients who show an autonomic imbalance with more prominent parasympathetic and less prominent sympathetic cardiac modulation already before Fingolimod-initiation are at increased risk of more pronounced HR slowing upon Fingolimod-initiation [11].

Since MS does not afflict the peripheral autonomic nervous system, and instantaneous HR-adjustment as well as HR counter-regulation depend on the integrity of the central autonomic network (CAN) [13], central autonomic dysfunction very likely accounts for prolonged or more pronounced, though mostly harmless [6] HR slowing upon Fingolimod-initiation. Many CAN structures are located in close proximity to brain areas typically involved in MS pathology [14]. Therefore, MS-related pathology may compromise timely and sufficient counter-regulation of Fingolimod-induced HR slowing and may thus result in more prominent HR slowing or delayed HR recovery[13].

We therefore hypothesize that autonomic dysfunction, such as the sympathetic-parasympathetic imbalance observed by Rossi et al., and the resulting, mostly harmless [6] Fingolimod-induced bradycardia [11] are due to pathophysiology of the central control and adjustment of cardiovascular autonomic modulation.

We therefore evaluated whether standard autonomic cardiovascular testing indicates central autonomic cardiovascular dysfunction prior to Fingolimod-initiation in those MS patients who need more than six hours after Fingolimod-initiation to re-increase their HR.

## Patients and Methods

From November 2012 to July 2013, we enrolled patients with relapsing-remitting MS in the study. We monitored cardiovascular autonomic modulation at rest and during autonomic challenge maneuvers, immediately before Fingolimod-initiation in the patients and compared results to those of age-matched healthy volunteers. As recommended by the European Medicines Agency, we continuously monitored HR in the MS patients after Fingolimod-initiation for at least six hours or longer if HR was at the lowest value 6 hours after the first dose (http://www.ema.europa.eu/ema).

Our study was approved by the Ethics Committee of the University of Erlangen-Nuremberg, and registered at the German Clinical Trial Register (DRKS00004548). All study participants gave written informed consent according to the declaration of Helsinki.

Patients who had received previous disease modifying treatments (DMT) were taken off their previous medication for at least the period consistent with current recommendation [15], i.e. for at least 6 months if the patients were on cytotoxic drugs (e.g. mitoxantrone), for at least 2–3 months if the patients were on natalizumab. Fingolimod could generally be started immediately after discontinuation of an interferon or glatiramer acetate.

To assure that we only tested patients in whom any possible finding of autonomic dysfunction could not be ascribed to other causes than the hypothesized dysfunction of the central autonomic network, we excluded participants from the study with any other diseases than MS or risk factors affecting the autonomic nervous system or clinically overt signs of autonomic dysfunction as well as participants on any medication possibly affecting the autonomic function. Furthermore, we excluded MS patients with high degrees of disability, such as spasticity, severe motor impairment or prominent sensory loss, as we had to assure that all study participants were able to adequately cooperate in autonomic challenge maneuvers such as the sustained handgrip (SHGE) exercise or the Valsalva maneuver (VM). All patients received their first dosage of Fingolimod within the hour after we had ended the autonomic tests. Participants were tested between 9 AM and 2 PM, after a resting period of at least 40 min that ensured a stable cardiovascular situation. To standardize study conditions, autonomic testing as well as HR monitoring was performed in a quiet room with an ambient temperature of 24°C and stable humidity. Study conditions were standardized throughout the entire observational period over 6 hours, and beyond in patients who required extended monitoring according to European Medicines Agency recommendations (http://www.ema.europa.eu/ema). None of our patients had any electrocardiogram (ECG) abnormalities prior to Fingolimod-initiation.

To estimate MS severity, we used the Expanded Disability Status Scale (EDSS) [16] and the Multiple Sclerosis Functional Composite (MSFC) [17].

To assess cardiovascular autonomic modulation, we recorded HR as RR-intervals (RRI; [ms]) by 3-lead electrocardiography, beat-to-beat systolic and diastolic blood-pressure (BPsys, BPdia; [mmHg]) by finger-pulse photoplethysmography (Portapress; TPD Biomedical Instrumentation, Amsterdam, The Netherlands), and respiratory frequency (RESP [min^-1^]) by chest impedance measurements [18,19], during three minutes at supine rest, and during autonomic challenge by active standing-up, metronomic deep breathing (MDB), during three Valsalva maneuvers (VM), and a three minute sustained-handgrip exercise.

Data were sampled, digitized and displayed on a personal computer and a custom designed data acquisition and analysis system (SUEmpathy, SUESS-Medizintechnik GmbH, Germany) and stored for off-line analysis [18]. From five-minute recordings without artifacts at rest, we extracted the most stationary 90-second epochs, then calculated mean values and standard deviations (SD) of all signals.

### Autonomic parameters

At rest and upon standing, we determined RRI-SD and the coefficient of variation of RRIs (RRI-CV), both reflecting sympathetic and parasympathetic cardiac modulation [19,20]. We calculated the square root of the mean squared differences of successive RRIs (RMSSD) reflecting parasympathetic cardiac modulation [19,20].

At rest and during standing, we also assessed sympathetic and parasympathetic modulation of RRI and BP by trigonometric regressive spectral analyses (TRS) of slow, underlying RRI- and BP-oscillations in the so-called low-frequency (LF; 0.04–0.14 Hz) and high-frequency (HF; 0.15–0.50 Hz) ranges reflecting autonomic RRI- and BP-modulation [18–21].

LF-oscillations of RRI at rest reflect sympathetic outflow and, to an undetermined degree, also parasympathetic activity; LF-oscillations of BP are related to sympathetic outflow only [19,20]. HF-oscillations of RRIs reflect parasympathetic activity [19,20], whereas BP-fluctuations in the HF-range are primarily a mechanical consequence of respiration-induced fluctuations in venous return and cardiac output [19,20]. The magnitude of LF- and HF-oscillations was determined as the integral under the power spectral density curves of RRI (ms^2^/Hz) and BP (mm Hg^2^/Hz) for the LF- and HF-frequency bands, and was expressed as LF- and HF-powers of RRI (ms^2^) and BP (mm Hg^2^) [18,21].

### Autonomic challenge maneuvers

#### Active standing

To assess cardiovascular and autonomic adjustment to orthostatic challenge, we asked all participants to stand up after a 10 minute resting period in supine position and to remain standing for three minutes [19]. We calculated changes in the above bio-signals and autonomic parameters, and additionally determined the 30/15-RRI-ratio [19], i.e. the ratio between the shortest RRI after approximately 15 heart beats and the longest RRI after approximately 30 heart beats upon standing-up.

#### Metronomic deep breathing (MDB)

During MDB, patients were asked to breathe deeply for three minutes at six cycles per minute, as this frequency normally produces maximal HR-increase during inspiration and decrease during expiration [19]. We calculated the ratio between the longest RRI during expiration and the shortest RRI during inspiration, i.e. the E/I-ratio [19].

#### Sustained handgrip exercise (SHGE)

Prior to the actual SHGE, participants were asked to press a handgrip dynamometer (Hydraulic Hand Dynamometer, MSD Europe, Londerzeel, Belgium) with their full strength. Then, they maintained the handgrip for three minutes at one-third of their individual maximum contraction strength. This exercise increases sympathetic activity and raises HR and BP [19,22]. The sympathetic activation depends on central command [23] and is buffered by CAN structures controlling baroreflex sensitivity [23,24].

We calculated mean values of Resp, RRI, BPsys, and BPdia during the last 15 seconds of exercise, and also assessed differences between the highest BPsys and BPdia values during handgrip and 60 second average values at rest [19].

#### Valsalva maneuver (VM)

During three VMs, participants maintained 40 mmHg expiratory pressure for 15 seconds [19]. In healthy persons, the sudden increase of intra-thoracic pressure causes a mainly mechanically mediated brief rise in BP and brief fall in HR (VM-phase I) [19,25]. The ongoing strain (VM-phase II) lowers venous cardiac return which reduces cardiac output and lowers BP (VM-phase II_early_). This BP-decrease induces baroreflex-mediated central cardiovagal inhibition and sympathetic activation resulting in a continuous HR-increase during VM-phase II, and peripheral vasoconstriction with BP increase during the VM-phase II_late_ [19,25]. With strain-release, BP briefly decreases while HR increases for 2–4 heart beats (VM-phase III) [19,25]. Yet, persistent vasoconstriction and re-increased cardiac output generate a BP-overshoot after strain-release [19,25]. This BP-overshoot causes baroreflex-mediated cardiovagal activation with HR-slowing during VM-phase-IV [19,25].

We determined minimal or maximal values of RRIs, BPsys, and BPdia during VM-phases I, II_late_, III, and IV, lowest BP values during VM-phase II_early_, and highest RRIs after strain-release. We also calculated the absolute and relative VM-phase-IV BP-overshoot as differences between maximum VM-phase-IV BPsys and BPdia values and baseline BP values prior to the VM, [as ΔBP = BP_IV_-BP_rest_ and as relative ΔBP = (BP_IV_-BP_rest_) / BP_rest_.].

We assessed the Valsalva-ratio, i.e. the ratio between the highest and lowest RRIs during the first 30 seconds after strain-release as index of the baroreflex-induced, parasympathetically mediated bradycardia [19,25]. Finally, we calculated the BP-related cardiovagal response after strain-release as ratio between the difference of the highest and lowest RRIs after strain-release (RRI_max_-RRI _min_) and the BP increase from baseline to maximum VM-phase-IV values, i.e. the BP increase which is the driving force of the post-strain cardiovagal HR slowing [ΔBP-related cardiovagal response = (RRI_max_-RRI_min_) / (BPsys _IV_-BPsys _rest_)] [19,25].

### Statistical Analysis

Data were tested for normal distribution using the Shapiro-Wilk test. We assessed differences in bio-signals and autonomic parameters at rest and during autonomic challenge between patients without and with prolonged HR-slowing (beyond 6 hours) upon Fingolimod-initiation and controls by analysis of variance for repeated measurements (ANOVA, general linear model). We used ‘challenge’ (rest, active standing, MDB, VM, and SHGE) as within-subject factors and ‘group’ (patients with and without prolonged HR-slowing upon Fingolimod-initiation, and controls) as between-subject factor. Suitability of the ANOVA model was assessed by Mauchly’s test of sphericity. In case of violation of the sphericity assumption, the Greenhouse Geisser correction was employed. In case of significant ANOVA results, we performed post-hoc single comparisons. For comparison of EDSS-, MSFC-scores, and the lowest HR-values upon Fingolimod-initiation between patients with and without prolonged HR-slowing, we used the Mann–Whitney U-test. We used the Chi-square test to assess gender differences between groups. Significance was set at p<0.05. For data analysis, we used a commercially available statistical program (IBM SPSS Statistics for Windows, Version 20.0. Armonk, NY, USA).

## Results

**Fig 1:**
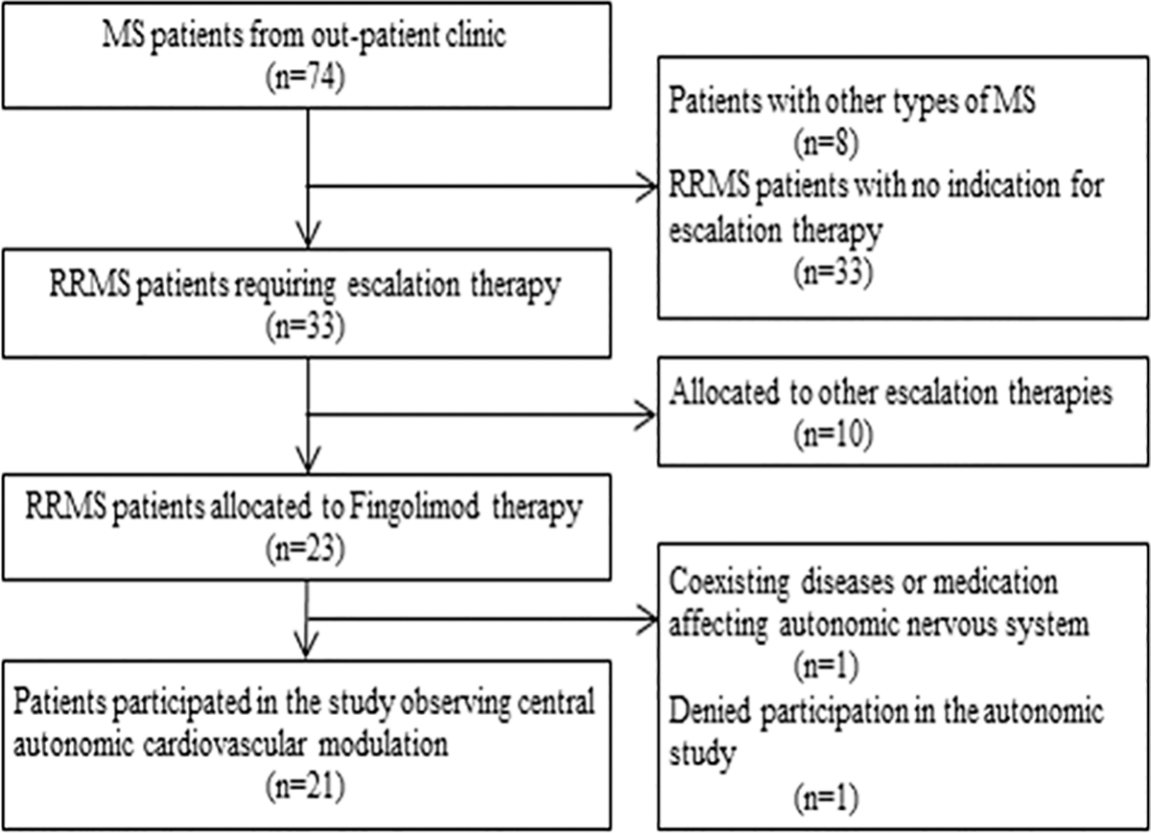
Flowchart of study patients.

21 patients (12 women; mean age 34.4 years, standard deviation [SD] 6.6 years) with relapsing-remitting MS participated in the study (Fig 1). Cardiovascular autonomic modulation at rest and during autonomic challenge maneuvers, immediately before Fingolimod-initiation was compared to that of 20 age-matched healthy volunteers (10 women; mean age, 30.1±12.5 years). Table 1 summarizes demographic data of patients and healthy persons.

**Table 1:** Demographic and clinical characteristics of multiple sclerosis patients without and with prolonged heart rate slowing (beyond 6 hours) upon Fingolimod-initiation and healthy persons.

	Patients without prolonged HR-slowing	Patients with prolonged HR-slowing	Healthy persons	*P-*value
Number of patients	14	7	20	
Sex [female/male]	9/14	3/7	10/10	> 0.05
Age [year]	34.2±6.5	34.9±7.2	30.1±12.5	> 0.05
Weight [kg]	71.2±18.2	74.2±17.5	72.5±3.5	> 0.05
Height [cm]	169.6±7.6	180.3±12.4	179.5±12.0	> 0.05
EDSS	2.0 (1.5–2.5)	2.0 (1.5–3.0)		> 0.05
MSFC	0.07 (-0.41–0.40)	0.41 (-0.17–0.61)		> 0.05
EDSS change from last year	0.0 (0.0–0.5)	0.0 (0.0–0.5)		> 0.05
Number of relapses in previous year	1 (1–2)	1 (1–2)		> 0.05
Interval in years from symptom- onset to date of examination	5.9 (3.5–11.2)	6.4 (3.0–14.1)		> 0.05
MS medication history [number of patients]				> 0.05
Interferon beta	9	2		
Natalizumab	2	3		
Glatiramer acetate	3	1		
Mitoxantrone	0	1		

Abbreviations: EDSS = Expanded Disability Status Scale; HR = heart rate; MSFC = Multiple Sclerosis Functional Composite. Age, weight and height are continuous variables with normal distribution, expressed as mean ± SD. MSFC and interval are continuous variables with non-normal distribution, and EDSS, and Change in EDSS were categorical variables, expressed as median (range).

**Fig 2:**
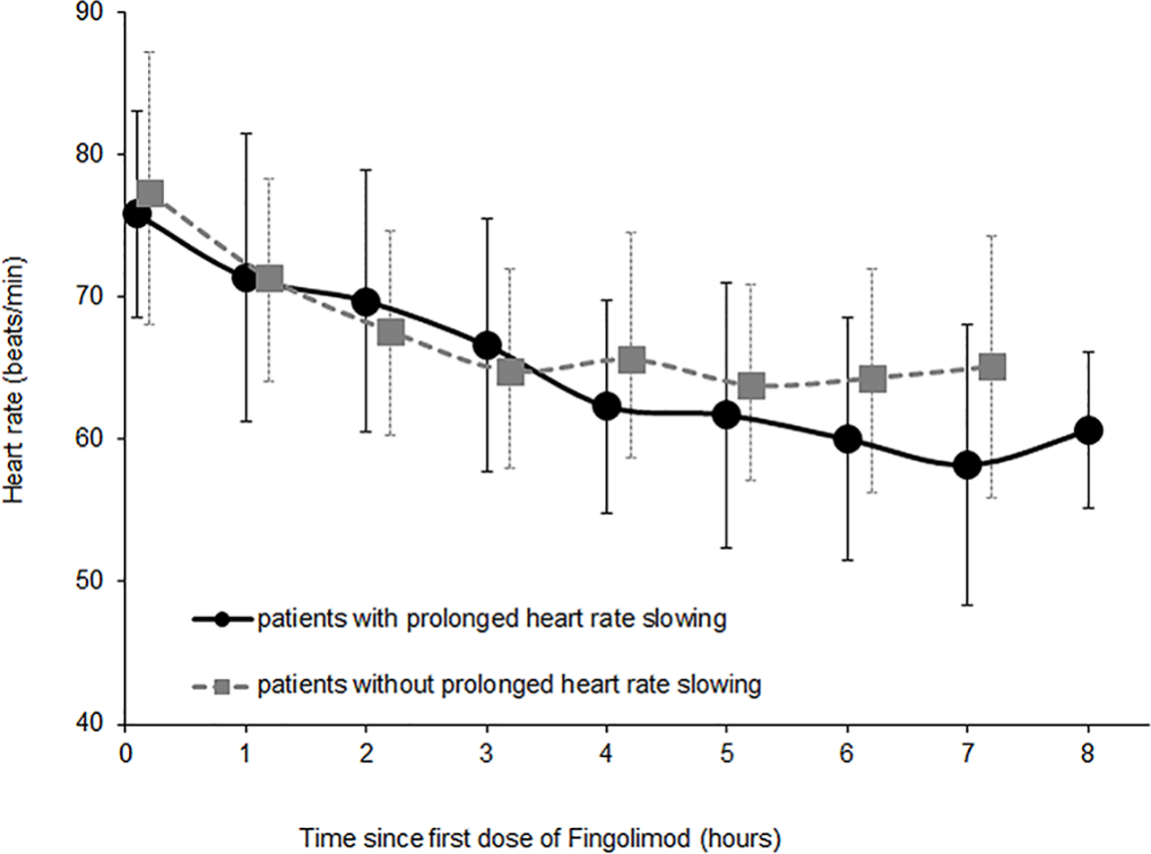
Mean heart rate immediately before Fingolimod-initiation and during 8 hours of monitoring after Fingolimod-initiation in MS patients with (n = 7) and without (n = 14) prolonged HR slowing.

Upon Fingolimod-initiation, all 21 MS patients showed HR-slowing (Fig 2), but no patient suffered from arrhythmia or atrioventricular blockage. None of the patients developed any ECG changes during or at the end of the first Fingolimod dosing, and all patients remained clinically asymptomatic. In 14 patients, HR re-increased within 6 hours, while seven patients had HR-slowing for more than 6 hours, and only re-increased HR within 8 hours. Patients with prolonged HR-slowing had lowest HRs (52.5 ± 5.1 beats/min) after 6 hours while patients without prolonged HR-slowing had lowest HRs (56.5 ± 6.7 beats/min) after 5 hours. HRs at rest, before Fingolimod intake, and the lowest HRs after Fingolimod intake did not differ between both patient groups (*p* > 0.05).

Clinical MS severity, as assessed by EDSS and MSFC values, was similar in patients with and without prolonged HR-slowing upon Fingolimod-initiation (EDSS: 2.0 (1.5–3.0) vs. 2.0 (1.5–2.5); MSFC: 0.41 (-0.17–0.61) vs. 0.07 (-0.41–0.40); *p* > 0.05; Table 1).

### Comparison between groups at rest

At rest, RRIs were significantly lower; i.e. HR was faster, in MS patients with and without prolonged HR-slowing than in healthy participants (*p* < 0.05; Table 2) but RRIs did not differ between the two patient subgroups. In contrast, resting BPsys and BPdia were significantly higher in the seven patients who needed more than 6 hours to re-increase HR from slowed values after Fingolimod-initiation than in the other 14 patients or the healthy persons (*p* < 0.05; Table 2).

**Table 2:** Mean values and SDs of bio-signals and time-domain parameters of RRI-variability at rest, upon standing-up, during metronomic deep breathing at six cycles/minute, and upon sustained handgrip exercise prior to Fingolimod-initiation in MS patients without and with prolonged heart rate slowing (for more than 6 hours) upon Fingolimod-initiation, and in healthy persons.

Parameters		14 pats. without prolonged HR-slowing [1]	7 pats. with prolonged HR-slowing [2]	20 healthy persons [3]	Comparison of groups		
					[1] vs. [2]	[1] vs. [3]	[2] vs. [3]
					*P-*value		
Resp [cycles/min]	supine rest	14.3±3.4	14.4±4.1	14.1±2.7		> 0.05	
	standing	14.3±4.1	16.7±3.5	13.7±3.7		> 0.05	
	metronomic deep breathing	6.1±0.4*	6.2±0.3*	6.5±0.7*		> 0.05	
	sustained handgrip exercise	16.3±4.3	13.8±4.8	14.3±4.8		> 0.05	
RRI [ms]	supine rest	786.8±93.6	797.6±75.3	893.7±119.9	>0.05	**0.006**	**0.010**
	standing	690.6±82.1*	652.3±97.5*	699.3±95.3*		> 0.05	
	metronomic deep breathing	806.2±96.1	809.1±68.2	883.7±141.8		> 0.05	
	sustained handgrip exercise	746.0±84.5*	695.3±123.5*	766.4±122.9*		> 0.05	
BPsys [mmHg]	supine rest	117.0±10.9	136.3±20.7	121.3±11.5	**0.003**	> 0.05	**0.014**
	standing	120.7±12.7	132.7±16.1	130.0±16.3		> 0.05	
	metronomic deep breathing	117.0±16.1	127.6±16.4	127.2±18.3		> 0.05	
	sustained handgrip exercise	131.8±14.4*	156.8±19.7*	139.5±15.1*	**0.001**	> 0.05	**0.016**
BPdia [mmHg]	supine rest	60.8±9.4	77.6±9.1	64.2±8.4	**<0.001**	> 0.05	**0.001**
	standing	65.9±11.5	73.0±11.0	65.5±9.0		> 0.05	
	metronomic deep breathing	56.5±9.6	62.6±16.8	64.5±8.3		> 0.05	
	sustained handgrip exercise	72.9±10.5*	92.6±16.3*	77.8±9.0*	**<0.001**	> 0.05	**0.004**
SD-RRI [ms]	supine rest	38.6±19.8	47.5±17.7	47.4±20.0		> 0.05	
	standing	33.5±9.1	38.7±16.1	33.4±14.3*		> 0.05	
CV-RRI [%]	supine rest	4.9±2.3	6.0±1.9	5.3±2.0		> 0.05	
	standing	5.0±1.5	5.9±2.0	4.5±1.7		> 0.05	
RMSSD [ms]	supine rest	29.9±18.8	37.8±25.0	44.5±25.1		> 0.05	
	standing	15.0±3.2*	22.7±11.8	16.8±7.4*		> 0.05	
30/15-ratio	standing	1.29±0.20	1.30±0.26	1.50±0.30		> 0.05	
Max BPdia-BPdia rest [mmHg]	sustained handgrip exercise	18.58±10.3	32.85±15.4	20.19±12.4	**0.013**	>0.05	**0.016**
E/I-ratios	metronomic deep breathing	1.34±0.16	1.46±0.21	1.48±0.20		>0.05	

Abbreviations: BPdia = diastolic blood pressure; BPsys = systolic blood pressure; CV = coefficient of variation; E/I-ratio = ratio between longest RRI during expiration and shortest RRI during inspiration; HR = heart rate, RESP = respiratory frequency; RMSSD = root mean square of successive differences; RRI = RR-intervals; SD = standard deviation. Significant differences between values at supine rest and values during standing, metronomic deep breathing or sustained handgrip exercise are indicated by an asterisk (*). Significant differences between groups are highlighted as **bold numbers**.

The other resting parameters, i.e. respiratory frequency, SD-RRI, CV-RRI, RMSSD, RRI-LF-powers, RRI-HF-powers, BP-LF-powers, BP-HF-powers, did not differ significantly between patients with and without prolonged HR-slowing and healthy persons (*p* > 0.05; Tables 2 and 3).

**Table 3:** Mean values and SDs of frequency domain parameters of RR intervals and blood pressure at supine rest and upon standing-up prior to Fingolimod-initiation in MS patients without and with prolonged heart rate slowing (for more than 6 hours) upon Fingolimod-initiation, and in healthy persons.

Parameters		14 pats. without prolonged HR-slowing [1]	7 pats. with prolonged HR-slowing [2]	20 health persons [3]	Comparison of groups		
					[1] vs. [2]	[1] vs. [3]	[2] vs. [3]
					*P-*value		
RRI-LF power [ms^2^]	supine rest	1089.7±1162.3	1376.8±934.8	1468.5±1363.2		> 0.05	
	standing	946.9±512.5	1326.1±1340.7	893.2±721.8		> 0.05	
RRI-HF power [ms^2^]	supine rest	437.1±429.2	747.8±943.3	273.7±1691.9		> 0.05	
	standing	77.9±50.7	281.1±254.9	115.2±90.2	**0.001**	> 0.05	**0.004**
BP-LF power [mmHg^2^]	supine rest	18.0±13.4	18.4±14.5	17.4±11.6		> 0.05	
	standing	35.3±25.8	57.1±44.4	28.8±21.5		> 0.05	
BP-HF power [mmHg^2^]	supine rest	4.4±3.6	5.0±3.4	3.9±2.5		> 0.05	
	standing	6.3±3.3	10.9±7.3	7.2±6.0		> 0.05	

Abbreviations: BP = blood pressure; HF = high frequency; HR = heart rate, LF = low frequency; RRI = RR-intervals; SD = standard deviation. Significant differences between groups are highlighted as **bold numbers**.

### Comparison between groups during standing-up

*Upon standing-up*, all groups significantly decreased RRIs, i.e. increased their HR, from resting values (*p* < 0.05; Table 2). In contrast, parasympathetic RMSSD values and RRI-HF-powers only decreased in the healthy controls and the 14 patients who re-increased their HR after Fingolimod-initiation within 6 hours. RMSSDs and RRI-HF-powers remained unchanged in the seven patients who re-increased HR after more than 6 hours post Fingolimod-initiation. Moreover, SD-RRI decreased significantly upon standing-up in healthy persons only but not in the MS patients.

In the three groups, values upon standing-up were similar for all other parameters, i.e. respiratory frequency, RRIs, BPsys, BPdia, SD-RRI, CV-RRI, RMSSD, RRI-LF-powers, BP-LF-powers, BP-HF-powers, and 30/15-RRI-ratios (*p* > 0.05; Tables 2 and 3). BPsys and BPdia did not change significantly upon standing-up. However, the patients who had HR-slowing beyond 6 hours upon Fingolimod-initiation slightly (*p* = 0.08) decreased BPsys by 6.2±18.8 mmHg and BPdia by 5.0 ± 17.7 mmHg upon standing-up, while the other patients and the healthy participants slightly increased BPsys and BPdia upon standing-up.

### Comparison between groups during sustained handgrip exercise and metronomic breathing

During sustained handgrip exercise (SHGE), all groups significantly accelerated HR, i.e. decreased RRIs, and increased BPsys and BPdia from baseline values (Table 2).

BPsys and BPdia averaged during the last 15 seconds of exercise were significantly higher in the seven patients with prolonged HR-slowing upon Fingolimod-initiation (156.8±19.7 mmHg; 92.6±16.3 mmHg) than in the 14 other patients (131.8±14.4 mmHg; 72.9±10.5 mmHg) or the healthy participants (139.5±15.1 mmHg; 77.8±9.0 mmHg).

In addition, the increase in BPdia from values at rest to the maximum BPdia value at the end of exercise was significantly higher in the seven patients with prolonged HR-slowing than in the other 14 patients and the healthy controls (Table 2).

During *MDB*, respiratory frequency, RRIs, BPsys, BPdia, E/I-ratios were similar between the three groups (*p* > 0.05; Table 2).

### Comparison between groups during Valsalva maneuvers

During *Valsalva maneuver*, maximal or minimal RRIs of VM-phases I, II, III, and IV, as well as the Valsalva-ratios were similar in patients with and without prolonged HR-slowing after Fingolimod-initiation and in healthy persons (*p* > 0.05; Table 4).

**Table 4:** Mean values and SDs of bio-signals and calculated autonomic parameters during Valsalva maneuver prior to Fingolimod initiation in multiple sclerosis patients without and with prolonged heart rate slowing (beyond 6 hours) upon Fingolimod-initiation, and in healthy persons.

Parameters	14 pats. without prolonged HR-slowing [1]	7 pats. with prolonged HR-slowing [2]	20 healthy persons [3]	Comparison of groups		
				[1] vs. [2]	[1] vs. [3]	[2] vs. [3]
				*P-*value		
**VM-phase I**						
max BPsys [mmHg]	140.6±17.0	144.7±25.1	168.8±23.4	> 0.05	**0.001**	**0.016**
max BPdia [mmHg]	79.3±11.1	76.6±17.0	91.8±13.5	> 0.05	**0.011**	**0.014**
**VM-phase II early**						
max RRI [ms]	835.4±113.0	893.0±153.4	934.6±182.7	> 0.05		
min BPsys [mmHg]	88.5±30.6	87.3±15.7	113.8±17.8	> 0.05	**0.003**	**0.012**
min BPdia [mmHg]	55.5±17.9	54.1±10.8	69.3±8.7	> 0.05	**0.004**	**0.011**
**VM-phase II late**						
min RRI [ms]	632.7±95.7	607.1±85.0	629.1±128.5	> 0.05		
max BPsys [mmHg]	109.7±31.7	118.0±16.6	149.3±24.2	> 0.05	**< 0.001**	**0.001**
max BPdia [mmHg]	68.6±19.7	75.8±11.8	94.5±14.3	> 0.05	**< 0.001**	**0.012**
**VM-phase III**						
min RRI [ms]	617.7±89.8	569.1±46.8	625.8±119.2	> 0.05		
min BPsys [mmHg]	92.1±28.4	87.9±26.8	117.0±22.1	> 0.05	**0.007**	**0.012**
min BPdia [mmHg]	57.8±17.3	55.2±14.9	70.8±9.4	> 0.05	**0.009**	**0.012**
**VM-phase IV**						
max RRI [ms]	1058.6±299.3	1049.7±258.4	1070.9±180.8	> 0.05		
max BPsys [mmHg]	171.2±23.0	149.8±20.8	179.4±19.2	> 0.05	> 0.05	**0.003**
max BPsys IV-BPsys rest [mmHg]	54.2±19.6	13.1±34.4	51.0±24.0	**0.001**	> 0.05	**0.001**
(max BPsys IV-BPsys rest) **/** BPsys rest	0.46±0.18	0.10±0.28	0.41±0.20	**0.001**	> 0.05	**0.002**
max BPdia [mmHg]	85.4±13.5	79.4±11.8	89.0±14.9	> 0.05		
max BPdia IV-BPdia rest [mmHg]	24.55±10.19	1.84±20.07	24.51±13.74	**0.001**	> 0.05	**0.001**
(max BPdia IV-BPdia rest) / BPdia rest	0.40±0.21	0.02±0.28	0.39±0.23	**0.001**	> 0.05	**0.001**
**Indices of baroreflex mediated cardiovagal buffer capacity**						
Valsalva ratio	1.7±0.4	1.9±0.3	1.8±0.4	> 0.05		
RRImax-RRImin **/** BPsysIV-BPsys rest	8.16±9.46	36.22±14.67	9.05±4.10	**<0.001**	> 0.05	**<0.001**

Abbreviations: BPdia = diastolic blood pressure; BPsys = systolic blood pressure; HR = heart rate; max = maximum; min = minimum; RRI = RR-intervals. Significant differences between groups are highlighted as **bold numbers**.

Maximum or minimum systolic and diastolic BP values of VM-phases I, II, and III were lower in patients than in healthy participants. Peak values during the VM-phase-IV BP-overshoot did not differ between groups for BPdia but were lower for BPsys in patients with prolonged HR-slowing after Fingolimod-initiation than in healthy participants.

The absolute and relative increases in systolic and diastolic BP-values from baseline, before VM, to the VM-phase-IV overshoot values [i.e. ΔBP = BP_IV_-BP_rest_ and ΔBP = (BP_IV_-BP_rest_) / BP_rest_] were significantly lower in patients with prolonged HR-slowing upon Fingolimod-initiation than in the other patients or the healthy participants (*p* < 0.05; Table 4).

The ratio reflecting the baroreflex mediated cardiovagal buffer-capacity in relation to the baroreflex input, i.e. the BP-increase from baseline to the maximum VM-phase-IV BP values was significantly, three to four times, higher in the patients with prolonged HR-slowing upon Fingolimod-initiation (36.22±14.67) than in the other patients (8.16±9.46) or healthy controls (9.05±4.1).

## Discussion

After the first dosage of 0.5 mg Fingolimod, all 21 MS patients slowed HR with a nadir after approximately 5 hours and lowest HR-values similar to those reported in healthy persons after Fingolimod [2,3,5,26,27].

Fingolimod slows HR due to mechanisms similar to those of parasympathetic activation [3,8].

Although pathways are different, vagal stimulation and Fingolimod activate the cardiac G protein-gated potassium channels [3] which results in negative chronotropic effects [3,8]. Similar to parasympathetically mediated HR-slowing, Fingolimod-related HR-slowing is reversed by atropine [3] or mitigated by alpha 1-receptor-agonists [8].

Although the Fingolimod induced HR-slowing occurs at the level of the heart [3,8], the HR-slowing in our MS patients after only 0.5 mg Fingolimod indicates an enhanced autonomic responsiveness [5] [11] to the chronotropic Fingolimod effects. This increased responsiveness is best ascribed to the central pathology of MS patients and most likely not caused by an augmented local responsiveness of their cardiac G protein-gated potassium channels to Fingolimod. Typically, MS lesions are centered around CAN structures such as the corpus callosum, peri- and paraventricular structures [28–31], and nuclei or pathways that modulate baroreflex sensitivity and cardiovascular autonomic function [13,32].

While 29.1% of the 55 MS patients studied by Rossi et al. developed significant bradycardia with HR below 50 bpm after Fingolimod-initiation [11] none of our patients had clinically relevant HR slowing upon Fingolimod-initiation. However, seven of our patients did not re-increase their HR within the six hours recommended as standard cardiovascular monitoring time after Fingolimod-initiation (http://www.ema.europa.eu/ema), but needed extended monitoring for up to eight hours. Similar to the patients studied by Rossi et al. [11], our seven patients showed signs of subtle, clinically non-overt autonomic cardiovascular dysfunction already prior to Fingolimod-initiation.

Theoretically, such subtle cardiovascular autonomic dysregulation might be due to dysfunction of the peripheral or the central autonomic nervous system. Yet, MS pathology is not associated with peripheral but with central autonomic nervous system pathology [33,34]. Most likely, the mild disturbances in cardiovascular responses to standard autonomic challenge manoeuvers are, therefore, caused by an interference of MS lesions with CAN structures that control cardiovascular function [13,32,35,36].

Similar to the increased parasympathetic activity observed by Rossi et al. prior to Fingolimod-initiation in those MS patients who developed prominent bradycardia upon Fingolimod-initiation [11], our seven patients with prolonged HR slowing upon Fingolimod showed increased parasympathetic activity upon standing–up already before Fingolimod therapy. Upon standing-up and unloading of the baroreceptors, the seven patients could not adequately reduce their parasympathetic HR modulation but they maintained their parasympathetically mediated RMSSD-values and RRI-HF-powers [19,20], at values similar to their supine parasympathetic modulation.

Central baroreflex control involves structures that are frequently afflicted by the neuro-inflammatory MS process [28–31], including the nucleus tractus solitarii and many rostral CAN areas [13], such as the magnocellular neurons of the supraoptic and paraventricular nuclei, posterior hypothalamus, paraventricular and dorsomedial hypothalamic nuclei, preoptic-anterior hypothalamic region, periaqueductal gray, central nucleus of the amygdala or the insular cortex [13], pathways to the dorsal vagal nucleus, nucleus ambiguous and rostral ventrolateral medulla [13,19,28–31].

It would have been desirable to identify a specific pattern of CAN-related MS-lesions in the seven patients with prolonged HR recovery upon Fingolimod-initiation. Yet, our patient group was too small to confirm an MRI-pattern of MS-lesions in the seven patients that clearly differed from the lesion-distribution or-size seen in the other 14 patients.

Still, MS-related CAN lesions very likely also account for the increased BP-overshoot during handgrip-exercise in the seven patients with delayed HR re-increase after Fingolimod-initiation. Normally, such BP-overshoot is prevented by central baroreflex structures that adjust baroreflex sensitivity [23] and mitigate sympatho-excitation during exercise to prevent sympathetic over-activity [23,24]. In contrast to the exercise-induced sympathetic overactivity seen in our patients with prolonged HR-slowing upon Fingolimod, Rossi et al. reported PR-time prolongation in their patients with bradycardia upon Fingolimod suggesting decreased sympathetic function [11]. The differences in central sympathetic cardiac control between both studies might be due to different locations of MS lesions and subsequently different changes in sympathetic and parasympathetic cardiovascular modulation.

Our findings during the Valsalva manoeuver further suggest a central baroreflex dysfunction in the seven MS patients who had a delayed HR re-increase after Fingolimod-initiation. In comparison to the other 14 patients and the healthy participants, the seven patients had less systolic BP-overshoot during VM-phase-IV and rather little BP increase from baseline to VM-phase-IV (Table 4). As the BP-overshoot during VM-phase-IV is due to a central, baroreflex mediated sympathetic activation in response to the strain-induced BP decrease in early VM- phase-II [19,23,25], we conclude that the seven MS patients had impaired central sympathetic adjustment and activation, again indicating an—MS related—impairment of central baroreflex modulation [13,19,23,28–31]. Although their BP-increase and overshoot during VM-phase-IV was rather small, the baroreflex mediated cardio-vagal HR slowing after strain-release was four times more prominent in the seven patients than in the other 14 patients or the healthy participants, as shown by the ratios between the difference of the highest and lowest RRIs after strain-release (RRI_max_-RRI _min_) and the BP-increase from baseline to maximum VM-phase-IV values (Table 4). A similar imbalance of sympathetic and parasympathetic cardiovascular modulation during the VM was observed by Rossi et al. in their MS patients [11]. The authors found a correlation between increased cardiovagal activity during the VM-phase-IV assessed prior to Fingolimod therapy and the nadir of HR slowing upon Fingolimod-initiation [11]. They suggested an association between the increase in parasympathetic modulation and negative chronotropic effects of Fingolimod [11]. Our finding of relatively exaggerated HR-slowing despite diminished BP-overshoot after VM-strain release suggest that central baroreflex-dysfunction causes the augmented parasympathetic responses after VM-strain release in those patients who have pronounced HR-slowing [11] or delayed recovery of HR upon Fingolimod-initiation.

In conclusion, none of our patients had clinically overt signs of autonomic dysfunction, clinically relevant HR-slowing upon Fingolimod-initiation, or developed arrhythmia, atrioventricular blockage, or other ECG changes upon Fingolimod-initiation; still, the results of autonomic testing prior to Fingolimod treatment, suggest that the seven patients who had a delayed HR re-increase upon Fingolimod-initiation have a subtle and clinically not overt central autonomic dysfunction.

Most likely, MS-related CAN lesions compromise autonomic adjustment to cardiovascular challenge and thus delay counter-regulation of HR-slowing upon Fingolimod-initiation.

We assume that more pronounced dysfunction of central autonomic control might also contribute to the pathophysiology of the rare cases of clinically relevant cardiac arrhythmias upon Fingolimod-initiation.

Our results suggest that a battery of autonomic challenge maneuvers—including baroreflex testing by orthostatic challenge, SHGE, and a VM—may identify patients at risk for prolonged or pronounced slowing of HR already prior to Fingolimod-initiation. Abnormal cardiovascular responses to these autonomic challenge maneuvers might refine the decision whether Fingolimod treatment should be initiated or whether patients need special cardiovascular monitoring during Fingolimod-initiation.

According to our results, the increased BP-overshoot during SHGE and the increased ratios between the difference of the highest and lowest RRIs after strain-release (RRImax–RRI min) and the BP-increase from baseline to maximum VM-phase-IV values might serve as potential surrogate markers predicting prolonged HR re-increase after Fingolimod-initiation. However, only further studies of large groups of MS patients who do not have any clinically overt autonomic dysfunction might confirm whether the above autonomic challenge maneuvers may be adequately sensitive to set up recommendations and guidelines for Fingolimod treatment.

While our patient groups were too small to calculate the predictive power of autonomic test results regarding prolonged or prominent HR slowing upon Fingolimod-initiation, subsequent multicenter studies might reach sample sizes that are required to calculate the predictive power of autonomic cardiovascular testing prior to Fingolimod-initiation.

As a further limitation, our study lacks a control group of MS patients who were on placebo or no therapy. However, Fingolimod has been an approved option for MS therapy since 2010 in the USA and Russia, and since 2011 in Switzerland and the European Union. Thus, it cannot be medically or ethically justified, not to provide adequate treatment to patients who are eligible for Fingolimod therapy.

Yet, further studies, comparing cardiovascular autonomic modulation of MS patients on Fingolimod and patients on other disease modifying treatment might be helpful to further strengthen our results.

## Supporting Information

S1 Protocol Study protocol. Investigator Initiated Study. Prospective observational study assessing relevance of autonomic nervous system testing and correlations between autonomic dysfunction and disease progression and severity in patients with relapsing remittent Multiple Sclerosis.
(PDF)

S1 TREND Checklist TREND Checklist of the present study. (DOC)
