# Supplementary material for: Central Autonomic Dysfunction Delays Recovery of Fingolimod Induced Heart Rate Slowing
Source: PLoS One. 2015 Jul 6;10(7):e0132139. doi: 10.1371/journal.pone.0132139 (PMC4493029; doi:10.1371/journal.pone.0132139)
Supplement: S1 Protocol — Investigator Initiated Study. Prospective observational study assessing relevance of autonomic nervous system testing and correlations between autonomic dysfunction and disease progression and severity in patients with relapsing remittent Multiple Sclerosis. (PDF) [file pone.0132139.s001.pdf]

Investigator Initiated Study

Prospective observational study assessing relevance of autonomic nervous system testing and correlations between autonomic dysfunction and disease progression and severity in patients with relapsing remittent Multiple Sclerosis

December 16, 2011

Principal investigator:

Prof. Dr. med. Dr. med. habil. Max J. Hilz
Dept. of Neurology
University of Erlangen-Nuremberg
Schwabachanlage 6
D-91054 Erlangen, Germany
Phone: +49-9131-8534444
Fax: +49-9131-8534328
e-mail: max.hilz@uk-erlangen.de

Co-Investigators:

Dr. med. Ralf Linker
Head of Multiple Sclerosis Unit
Dept. of Neurology
University of Erlangen-Nuremberg
Schwabachanlage 6
D-91054 Erlangen, Germany
Phone: +49- 9131- 8532187
Fax: +49- 9131- 8534545
e-mail: ralf.linker@uk-erlangen.de

NN

Dr. med. Julia Köhn
Dept. of Neurology
University of Erlangen-Nuremberg
Schwabachanlage 6
D-91054 Erlangen, Germany
Phone: +49- 9131- 8544320
Fax: +49- 9131- 8534328
e-mail: julia.koehn@uk-erlangen.de

Sebastian Möller
Dept. of Neurology
University of Erlangen-Nuremberg
Schwabachanlage 6
D-91054 Erlangen, Germany
Phone: +49- 9131- 8544545
Fax: +49- 9131- 8534328
e-mail: sebastian.moeller@uk-erlangen.de

NN

NN

Table of Contents:

I) Summary **p. 3**

II) Background **p. 9**

1) Clinical autonomic dysfunction necessitates autonomic function testing of various organs and systems p. 11

2) Pupillary light reflex responses p. 12

3) Disturbed saccadic eye movements as a predictor of MS severity and handicap p. 13

4) Cardiovascular autonomic dysfunction in MS p. 14

5) Bladder dysfunction in MS p. 15

6) Sexual dysfunction in MS p. 16

III) Rationale **p. 17**

IV) Ethics approval **p. 18**

V) Patient Population and Selection **p. 19**

VI) Investigational Plan and Methods **p. 21**

1) Enrollment of patients p. 21

2) Cross sectional and 36 months prospective evaluation of disease severity p. 22

2.1) Expanded Disability Status Scale (EDSS) p. 22

2.2) Multiple Sclerosis Functional Composite (MSFC) p. 22

3) Infrared Light reflex pupillography p. 24

4) Assessment of disturbed saccadic eye movements by Video-Nystagmography p. 26

5) Cardiovascular autonomic dysfunction p. 27

5.1) Monitoring of cardiovascular biosignals p. 27

5.2) Data processing p. 28

5.3 Autonomic bio-signal variability at rest p. 29

5.4) Heart rate and blood pressure responses and spectral powers of autonomic modulation during active standing p. 31

5.5) Assessment of baroreflex sensitivity p. 32

5.6) Blood pressure and heart rate responses during the Valsalva maneuver p. 33

5.7) Heart rate variation during 3-minute deep breathing p. 35

5.8) Blood pressure response to sustained handgrip test p. 36

5.9) Classification of autonomic cardiovascular function p. 36

6) Assessment of bladder function p. 37

6.1) Assessment of the urine status with dip stick p. 38

6.2) Post-voiding residual urine sonography p. 38

6.3) Uroflowmetry p. 39

7) Questionnaires assessing sexual dysfunction, bladder dysfunction and autonomic symptoms p. 41

VII) Statistical analysis **p. 42**

VIII) References **p. 43**

IX) Table: Procedures suited for non-invasively assessing correlations between clinical MS severity and autonomic nervous system dysfunction in a cross-sectional and prospective 36 months follow-up study of patients with preselected disease severity and treatment history **p. 50**

X) Timeline of Assessment **p. 52**

I) Summary

Autonomic nervous system (ANS) dysfunction seems to be common but is poorly defined in Multiple Sclerosis

Autonomic nervous system dysfunction has been frequently described in Multiple Sclerosis (MS) patients (Merkelbach et al., 2006) and may significantly affect quality of life of MS patients (Acevedo et al., 2000; Drory et al., 1995; Flachenecker et al., 1999; Linden et al., 1995; Nasseri et al., 1998; Vita et al., 1993).
MS is an inflammatory, demyelinating, neurodegenerative disorder of the central nervous system with unknown etiology (Stuve and Oksenberg, 1993). The onset peak ranges between the age of 20 and 40 years, and women are affected approximately twice as often as men. Most common clinical signs and symptoms in MS patients include sensory disturbance of the limbs, partial or complete visual loss, acute and subacute motor dysfunction of the limbs, diplopia, and gait dysfunction (Stuve and Oksenberg, 1993). However, MS lesions may involve brain areas which contribute to the central autonomic network (Benarroch, 1997b). Lesions of these brain areas may account for compromised adjustment of cardiovascular, sudomotor, inner organ and many other functions that are not under volitional but autonomic nervous system (ANS) control (Hilz, 2002; Low, 1997).
Therefore, dysfunction of the autonomic nervous system has been frequently reported in patients suffering from MS (Merkelbach et al., 2006), and may include bladder, bowel and sexual function, cardiovascular, sudomotor and pupillary function (Merkelbach et al., 2006). However, reports are heterogeneous (Acevedo et al., 2000; Drory et al., 1995; Flachenecker et al., 1999; Linden et al., 1995; Nasseri et al., 1998; Vita et al., 1993), mainly reflect function of singular organ systems (Acevedo et al., 2000; Drory et al., 1995; Flachenecker et al., 1999; Linden et al., 1995; Nasseri et al., 1998; Vita et al., 1993) at varying MS stages, and studies are mostly cross-sectional but not prospective (Acevedo et al., 2000; Drory et al., 1995; Flachenecker et al., 1999; Linden et al., 1995; Nasseri et al., 1998; Vita et al., 1993).

ANS dysfunction impairs quality of life and life expectancy and correlates with disease prognosis.

In common diseases such as diabetes mellitus, autonomic dysfunction is known to reduce quality of life and even life expectancy (Ewing et al., 1980; Low and Hilz, 2008). In diabetes, which is the most frequent etiology of autonomic dysfunction (Low and Hilz, 2008), occurrence of cardiac autonomic dysfunction is associated with significantly increased mortality rates (Ewing et al., 1980; Ewing and Clarke, 1987). Ewing and co-workers even reported a mortality rate of 44% at 2.5 years and 56% at 5 years in diabetic patients with symptomatic autonomic neuropathy (Ewing et al., 1980).
For example, impairment of the baroreflex is associated with a deterioration of prognosis in many diseases (Lanfranchi and Somers, 2002; Ormezzano et al., 2008; Parati et al., 2001; Sykora et al., 2009). The reflex arch depends on central autonomic network interaction and on modulation of

sympathetic and parasympathetic outflow towards heart and vasculature (Eckberg and Sleight, 1992; Hilz et al., 2011a; Hilz et al., 2010). Deterioration of the baroreflex sensitivity, i.e. the degree of heart rate adjustment to changes in blood pressure and vice versa (Eckberg and Sleight, 1992; Hilz et al., 2011a; Hilz et al., 2010), correlates with increased risk of cardiovascular complications and deteriorating prognosis, e.g. in arterial hypertension, heart failure, myocardial infarction, renal failure, or stroke (Lanfranchi and Somers, 2002; Ormezzano et al., 2008; Parati et al., 2001; Sykora et al., 2009), while improvement of baroreflex sensitivity, correlates with improved prognosis (Chan et al., 2005; Chan et al., 2008; Parati et al., 2001).

Attempt to establish associations between MS severity and ANS dysfunction requires readily available autonomic function tests.

For MS, there are - to our knowledge - no studies specifically correlating standard clinical parameters of cross-sectional disease severity and prospective changes in disease severity with parameters of autonomic nervous system function.

In order to improve the clinical evaluation and long-term monitoring of MS patients, a readily available standardized battery of autonomic tests is needed to identify and treat autonomic dysfunction at early stages and thus prevent secondary complications that unnecessarily deteriorate the patient's health status and prognosis.

Options to assess autonomic function are manifold (Hilz, 2002; Low, 1997) and comprise sophisticated procedures that are only available at specialized centers, such as scintigraphic assessments of autonomic cardiac innervation (Druschky et al., 1999; Hilz et al., 2003), orthostatic challenge tests by lower body negative suction paradigms (Marthol et al., 2007), or baroreflex testing by neck suction procedures (Eckberg and Sleight, 1992; Hilz, 2002; Hilz and Dutsch, 2006; Marthol et al., 2006).

Yet, many of these procedures are not suited for the widespread, easy and non-invasive application in daily routine examination of MS patients.

Given the potential negative effects of autonomic dysfunction on MS patients' quality of life and risk of mortality (Ewing et al., 1981; Lanfranchi and Somers, 2002), standardized, non-invasive, readily applicable autonomic testing procedures are needed to enable any MS treating neurologist - without specialized autonomic background or laboratory – to evaluate and monitor autonomic function in MS patients.

In this study, we therefore intend to determine whether an easily applied set of autonomic tests and questionnaires is suited to adequately assess autonomic dysfunction that may induce secondary complications such as cardiovascular, bladder, ocular or visual dysfunction, and to correlate such dysfunction with the clinical disease status.

Attempt to unveil associations between MS severity and ANS dysfunction requires selection of a rather homogeneous patient group

One limitation of any study evaluating suitability of an autonomic testing battery as an additional marker of MS course and severity arises from the high variability and inter-individual diversity of MS and its long-term course (Merkelbach et al., 2006).

To better standardize the patient population and limit disease heterogeneity, only patients with relapsing remitting MS and a predefined disease history should be enrolled in a study comparing autonomic function parameters with clinical or neuroradiological parameters of MS severity (Balcer, 2001).
Unless there is some likelihood of autonomic dysfunction at the onset of a study comparing ANS dysfunction and clinical MS severity, validity of any correlation between ANS dysfunction and parameters of MS severity remains uncertain.

Therefore patients at very early stages of disease without ANS symptoms might not be suited for a study seeking correlations between MS severity and ANS dysfunction.
Similarly, patients at highly advanced MS stages, e.g. with end-stage motor dysfunction, seem poorly suited for a study prospectively assessing the prognostic values of autonomic parameters since ANS function may still change while motor function is already at an end-stage steady-state without further deterioration.

Enrolment of MS patients with high disease activity despite treatment with β-interferon

To improve chances of finding associations between ANS dysfunction and MS severity in a cross-sectional patient population as well as a prospective follow-up comparison of autonomic and clinical findings, we therefore intend to enrol patients with relapsing-remittent MS at a rather well defined stage of therapy.
We shall include only patients who have already undergone disease modifying therapy with glatiramer acetate or interferone beta treatment but still experience disease progression and MS relapses.
Within the enrolled patient group, we shall conduct follow-up comparisons between autonomic parameters and clinical parameters of MS severity for a period of 36 months, to decide whether autonomic dysfunction correlates with disease development.
As common denominator of the MS patients to be enrolled in our study of correlations between MS severity and ANS dysfunction, we will ask patients for their participation in our evaluation who have a high disease activity under treatment with baseline therapies, i.e. patients who had at least one relapse in the previous year under therapy and who display at least 9 T2-hyperintense lesions or at least one gadolinium enhancing lesion in a cranial magnetic resonance imaging (MRI) study despite β-interferon or glatiramer acetate therapy. Additionally, untreated patients with severe, rapidly progressing, relapsing-remitting MS will be included, i.e. patients who had two or more relapses with progression of disability in the previous year and who display one or more gadolinium enhancing lesions ,or a significant increase of T2-hyperintense lesions compared to the previous cranial MRI. Typically, these are MS patients who are eligible for escalation treatment with Fingolimod or natalizumab.
Therefore, we will enrol patients in our study who have been offered an escalation therapy independently from our study and prior to our request for participation in the study evaluating correlations between MS course and severity and ANS dysfunction.

The decision of the primary neurologist to put a patient on Fingolimod (or natalizumab) treatment will be made independently from and prior to our asking the patient whether he or she would be willing to participate in the comparison of ANS dysfunction with clinical MS severity.
We will assess ANS function in comparison to clinical parameters of MS severity after the MS treating neurologist and the patient have agreed on the Fingolimod therapy and after the patient subsequently was informed about our study and has then given written informed consent to participate in our study.

Time course of comparisons of ANS function with clinical parameters of MS severity

To minimize the confounder of heterogeneous duration of disease and disease modifying therapy, and to assure that the time course of our correlations is comparable among the enrolled patients, we will determine ANS function and clinical diseases severity in all patients fulfilling enrolment criteria prior to the first Fingolimod treatment, within 24 hours after the first therapy, after 6 months ± 2 weeks upon enrolment, after 12 months ± 2 weeks upon enrolment, after 24 months ± 2 weeks upon enrolment, after 36 months ± 2 weeks upon enrolment.

Scoring clinical MS severity with the *Expanded Disability Status Scale* (EDSS), and the *Multiple Sclerosis Functional Composite*

To compare clinical MS severity with ANS function during our 3-year follow up study, we will use the *Expanded Disability Status Scale* (EDSS), assessing the degree of neurological impairment (Kurtzke, 1983) and the *Multiple Sclerosis Functional Composite* (MSFC), assessing leg, arm and cognitive function (Cutter et al., 1999).

Prospective assessment of autonomic pupillary, cardiovascular, bladder and sexual function in a rather homogeneous group of MS patients

Common tests used to evaluate ANS dysfunction in widespread diseases, such as diabetes mellitus, comprise assessment of autonomic cardiovascular function by means of heart-rate variability testing at rest, during deep breathing, active standing, during a Valsalva maneuver, and during the sustained handgrip test (Hilz and Dutsch, 2006), evaluation of the sympathetically and parasympathetically mediated pupillary light reflex responses (Dutsch et al., 2004), measurement of bladder function by uroflowmetry and residual volume assessments, evaluation of general autonomic function and sexual function by means of history taking or questionnaires (Suarez et al., 1999).

Pupillary light reflex impairment and impaired saccadic eye movements in MS

Pupillary abnormalities have rarely been evaluated in MS (de Seze et al., 2001a) although there is frequent impairment of pupillary function in MS patients. De Seze at al. reported that the parasympathetic system is most commonly affected, most likely linked to axonal loss rather than to demyelinating lesions (de Seze et al., 2001a). Furthermore, ocular motility disorders are

frequent in MS, but often remain under-diagnosed (Rougier and Tilikete, 2008). Eye movement disorders are mostly related to brain-stem and cerebellum lesions (Rougier and Tilikete, 2008). Light reflex pupillography and testing of horizontal saccadic eye movements are easily performed procedures assessing afferent as well as efferent branches of autonomic reflex arches (Hilz, 2002). In order to compare autonomic pupillary function with disease progression and severity, we will assess pupil size, light reflex amplitude as well as constriction and re-dilation velocities. Pupil size and re-dilation velocities are parameters reflecting sympathetic pupillary modulation (Heller et al., 1990; Smith, 1993; Smith and Dewhirst, 1986), while light reflex amplitude and constriction velocity are parameters depending on parasympathetic pupillary modulation (Heller et al., 1990; Piha and Halonen, 1994; Smith, 1993).

Cardiovascular autonomic dysfunction

Cardiovascular autonomic dysfunction has been reported to reach a prevalence of 10 to 50 % in MS patients (Merkelbach et al., 2006). Loss of sympathetic and parasympathetic control in the cardiovascular system has been reported, suggesting that demyelinating plaques may damage vasomotor centers in the brainstem or interfere with descending fibers of the autonomic nervous system in the spinal cord (Acevedo et al., 2000; Vita et al., 1993).

Determination of cardiovascular autonomic function is based upon a battery of different autonomic tests (Merkelbach et al., 2006). However, the pattern of pathological findings in MS is inconsistent and accounts for predominantly sympathetic involvement in some patients and predominantly parasympathetic pathology in others (Merkelbach et al., 2006).

In order to compare cardiovascular autonomic function with disease progression and severity, we will assess autonomic function by non invasive, readily performed procedures. We will assess heart rate and blood pressure responses and spectral powers of autonomic modulation at rest, during active standing, during the Valsalva maneuver, during 3-minute deep breathing, and during the sustained handgrip test.

Urinary tract infections and bladder dysfunction in MS

Urinary tract infections due to autonomic bladder dysfunction are among the most common causes for MS deterioration and death due to secondary complications (DasGupta and Fowler, 2002; Fernandez, 2002). Autonomic bladder dysfunction has significant impact on the extent of disability and impairment of daily activities of MS-patients (Nakipoglu et al., 2009). Voiding dysfunction is common in MS-patients and severely compromises quality of life and social interaction (Araki et al., 2002). Detrusor-hyporeflexia and detrusor-sphincter-dysynergia are indicative for pontine or spinal lesions in MS-patients (Araki et al., 2003). Disconnection of neuronal pathways between pons and sacral spine may account for reduced sphincter relaxation during detrusor contraction and lead to incomplete bladder emptying and dilatation of the upper urinary pathways with subsequent chronic renal failure (Brady and Fowler, 2001; Stewart and Fowler, 1999).

In order to compare autonomic bladder function with disease progression and severity, we will perform bladder ultrasonography to assess post-void residual urine volume (Kragt et al., 2004). In

addition, we will perform uroflowmetry to assess altered voiding profile (Brady and Fowler, 2001; Stewart and Fowler, 1999). In combination with a questionnaire, uroflowmetry and ultrasound will assess micturition urgency, bladder detrusor hyperreflexia, detrusor-sphincter dyssynergia, detrusor hyporeflexia. Urine dip stick analysis will be used to diagnose urinary tract inflammation that may arise from autonomic bladder dysfunction.

Sexual dysfunction in MS

There are limited data reflecting sexual dysfunction in MS-patients (Kirkeby et al., 1988; Lundberg, 1981). Previous studies report prevalence of erectile dysfunction in 60%, of ejaculatory dysfunction and/or orgasmic dysfunction in 50% and reduced sexual desire in 40% of MS patients (Lundberg, 1981).

Most female MS-patients report different types of sexual disorders (Kirkeby et al., 1988; Lundberg, 1981). Impairment of the spinal cord and of central autonomic structures contributing to sexual function is a major cause of erectile dysfunction (Betts et al., 1994; Zivadinov et al., 1999) .

In order to compare sexual autonomic function with disease progression and severity, we will use a battery of standardized as well as self-designed questionnaires to assess sexual dysfunction in male and female MS patients.

Autonomic nervous system questionnaire

A questionnaire will be used to assess the patient’s subjective autonomic symptoms and to compare the derived score of autonomic dysfunction with scores reflecting clinical disease progression and severity

II) Background

Involvement of central autonomic nervous system in MS pathology causes frequent autonomic dysfunction of various organs and reflex systems:
Autonomic dysfunction may be frequent in MS patients due to the MS pathophysiology and the involvement of the central autonomic nervous system in the disease process (Merkelbach et al., 2006).
Autonomic dysfunction of bladder, sexual organs, cardiovascular regulation, sweating, and inadequate sympathetic and parasympathetic adjustment of the iris and pupil to accommodation of changes in illumination (Egg et al., 2002; Jakobsen, 1990) are among the common autonomic symptoms of MS patients (Drory et al., 1995; Flachenecker et al., 1999; Haensch and Jorg, 2006; Kale et al., 2009; Linden et al., 1995; Nasseri et al., 1998).
In a transversal study of 40 patients, Acevedo et al. used a battery of cardiovascular autonomic tests, including postural blood pressure changes, postural heart rate changes, heart rate changes on inspiration and forced expiration and electrocardiographic R-R interval modulation during a Valsalva maneuver, and. found close correlations between cardiovascular autonomic dysfunction and the severity of MS as assessed by the expanded disability status scale (EDSS) (Acevedo et al., 2000). Several studies found similar correlations between progression of disability or disease severity in MS patients and impairment of cardiovascular autonomic function tests (Flachenecker et al., 2001; Gunal et al., 2002).

The wide-spread, complex central autonomic network involves basically the entire central nervous system (Benarroch, 1997a) and is almost inevitably compromised in MS patients with inflammatory or demyelinating lesions that very frequently involve areas closely involved in autonomic control, e.g. the areas close to the corpus callosum (Benarroch, 1997a), the hypothalamus, fornix, anterior commissure, internal capsule and optic system may compromise autonomic modulation in MS patients (Acevedo et al., 2000; Huitinga et al., 2001).
Imbalance of sympathetic and parasympathetic cardiovascular modulation due to central autonomic network involvement in the disease (Merkelbach et al., 2006) may cause relevant autonomic dysfunction in MS patients (Drory et al., 1995; Flachenecker et al., 1999; Linden et al., 1995; Nasseri et al., 1998).
There is some evidence that autonomic dysfunction correlates with MS progression and severity (Drory et al., 1995; Flachenecker et al., 1999; Linden et al., 1995; Nasseri et al., 1998).
Correlations between clinical MS severity and severity of autonomic dysfunction may be suited to improve clinical staging, therapeutic guidance and risk stratification in MS patients.

However, the course and progression of MS vary significantly among MS patients (Stuve and Oksenberg, 1993) and may thus compromise calculation of correlations or reduce the prognostic validity of correlations between autonomic nervous system dysfunction and clinical MS severity assessed in a cross-sectional, transversal study of MS patients due to the heterogeneity of disease stages or to the variability of effects of different disease modifying therapies.

Correlations between clinical MS severity and ANS dysfunction are likely to be more valid if the disease heterogeneity and inter-individual diversity of MS progression or variability of disease modifying approaches are limited by assessing the cross-sectional ANS dysfunction and clinical MS severity in patients at comparable stages of disease and therapy, and by prospectively recalculating correlations from follow-up assessments of several years.

In an attempt to better standardize the patient population and limit disease heterogeneity, only patients with relapsing remitting MS and a predefined disease history should be enrolled in a study with the objective to compare and correlate autonomic function parameters with clinical parameters of MS severity (Balcer, 2001).

Moreover, disease stages must be well defined to successfully determine associations of ANS dysfunction with clinical MS severity.

MS patients at very early stages may no yet manifest autonomic dysfunction; in contrast, MS patients at very advanced disease stages may be e.g. wheelchair-bound or bed-ridden, i.e. at stages of clinical impairment that do not progress much, while autonomic organ dysfunction might still progress. Both groups seem poorly suited for a study assessing cross-sectional and prospective correlations between the clinical status or course of MS severity and the degree of autonomic dysfunction.

To avoid flaws in a study correlating autonomic dysfunction with clinical MS severity, the disease inherent heterogeneity of severity and progression should be limited by excluding patients who are at early disease stages that might not manifest autonomic dysfunction as well as excluding patients with end-stage clinical impairment in whom changes in autonomic function might no longer correlate with changes in clinical severity.

Instead, validity of cross-sectional and prospective clinical and autonomic correlations may be improved by enrolling MS patients at more homogeneous stages of disease and pre-treatment history. These criteria seem to be more appropriately met by patients who have a high disease activity under treatment with baseline therapies, i.e. patients who had at least one relapse in the previous year under therapy and who display at least 9 T2-hyperintense lesions or at least one gadolinium enhancing lesion in a cranial magnetic resonance imaging (MRI) study despite β-interferon or glatiramer acetate therapy, or untreated patients with severe, rapidly progressing, relapsing-remitting MS will be included, i.e. patients who had two or more relapses with progression of disability in the previous year and who display one or more gadolinium enhancing lesions ,or a significant increase of T2-hyperintense lesions compared to the previous cranial MRI.

1) Clinical autonomic dysfunction necessitates autonomic function testing of various organs and systems

Autonomic disorders compromise the patient's quality of life significantly beyond the many aspects of impairment associated with MS; ANS dysfunction moreover poses risks of secondary complications that may shorten life expectancy of MS patients (Haensch and Jorg, 2006). Consequently; diagnosis of autonomic dysfunction and severity as well it progression may improve patient care and treatment options and will more rapidly identify risk of secondary MS complications, such as reduced cardiovascular adjustment, orthostatic dysregulation, bladder dysfunction with urinary tract infection, visual disturbances due to impaired pupillary adjustment, partnership problems due to sexual dysfunction, to name only few of the multiple complications secondary to autonomic dysfunction (Low, 1997).
Moreover, correlations between clinical MS severity and ANS dysfunction may improve prognostic evaluation and risk stratification in MS patients.

Autonomic dysfunction may involve many organ systems during the course of MS. Complications arising from ANS dysfunction are highly relevant at the level of pupillary adjustment mediated by central ANS structures and by cranial autonomic nerves (Dutsch et al., 2004; Hilz, 2002), at the cardiovascular system adjusting heart rate and blood pressure to instantaneously changing requirements, at the autonomic bladder innervation contributing to secondary urinary tract complications and inflammation, and at the level of sexual function affecting quality of life and partnership, particularly among younger MS patients still planning parenthood.
Validity of tests assessing correlations between the different types of ANS dysfunction and clinical MS severity must be determined in a rather homogeneous group of MS patients with similar stages of disease or treatment history.

2) Pupillary light reflex responses

In patients with relapsing forms of MS, the light reflex pathways are frequently involved in the disease process (Frohman et al., 2005).
Jakobsen reported prolonged latencies of the pupillary light response in MS patients, using television pupillography, and moreover saw close correlations of prolonged latencies with walking performance ($R = 0.76$; $p < 0.001$) (Jakobsen, 1990).
Pupillary light reflex testing non-invasively evaluates changes in disease pathology and function due to progression of structures involved in the pupillary light reflex (Hilz, 2002).

Pupillary function is under sympathetic and parasympathetic control (for review see (Hilz, 2002)). The interaction of both systems assures the spontaneous and light induced changes of pupillary diameter. Assessment of diameter changes in response to light stimuli non-invasively evaluates cranial autonomic function.
Light stimulation of retinal ganglion cells activates axons that travel along the optic nerve, partially cross the optic chiasma, bypass the lateral corpus geniculatum and reach the pretectal nuclear complex of both sides. The pretectal neurons activate the Edinger-Westphal nuclei of both sides and induce the parasympathetic pupillary constrictor response. The response is modulated by other structures of the central nervous system such as the cerebellum (Hilz, 2002).

Pupillary dilatation occurs during arousal and in darkness and depends on a central inhibition of the Edinger-Westphal nuclei as well as the activation of the peripheral sympathetic pathway (Lowenstein and Loewenfeld, 1950). Dilatation is also influenced by cortical areas such as frontal lobe areas (Hilz, 2002). The sympathetic pathway originates from the ipsilateral hypothalamus and descends through the subthalamus, midbrain and brainstem to the cervical spinal cord where it synapses in the ciliospinal center of Budge in the intermediolateral cell column at the level C8-T2 (Hilz, 2002).
We will use infrared pupillography, a non-invasive, readily performed method that quickly assesses functional changes due to MS related dysfunction in central autonomic network structures involved in the pupillary light reflex.
To better determine the clinical and prognostic value of altered pupillary light reflex parameters, we will evaluate the cross-sectional prevalence and severity as well as the prospective course of altered pupillary light reflex parameters in MS patients, and we will correlate results of pupillary light reflex measurements (for details, see below) with scores of clinical MS severity cross-sectionally at study onset and prospectively during a 36 months follow-up period in our predefined group of MS patients with the above and below mentioned enrollment criteria.

3) Disturbed saccadic eye movements as a predictor of MS severity and handicap

Neuro-ophthalmologic disorders with altered eye movements are common in MS but often under-diagnosed (Rougier and Tilikete, 2008). Mostly, ocular motility disorders of MS patients are related to brain-stem and cerebellum lesions (Eggenberger, 1996; Frohman et al., 2005; Rougier and Tilikete, 2008).

The incidence of eye movement disorders has been reported to vary between 60 % and 80 % of MS patients (Reulen et al., 1983). Eye movement disorders are even considered to be predictive of the severity of disease and handicap (Derwenskus et al., 2005; Serra et al., 2003).

Saccadic eye movements are high velocity eye movements that involve brainstem and cerebellar structures including the rostral interstitial nucleus of the median longitudinal fascicle for vertical saccades and the pontine paramedian reticular formation for horizontal saccades (Eggenberger, 1996; Frohman et al., 2005; Rougier and Tilikete, 2008).

Changes in saccadic eye movements may correlate with disease severity and progression (Derwenskus et al., 2005; Serra et al., 2003).

To better determine the clinical and prognostic value of altered saccadic eye movements, we will evaluate the cross-sectional prevalence and severity as well as the prospective course of altered saccadic eye movements in MS patients, and we will correlate results of saccadic eye movement recordings (for details, see below) with scores of clinical MS severity cross-sectionally at study onset and prospectively during a 36 months follow-up period in our predefined group of MS patients with the above and below mentioned enrollment criteria.

4) Cardiovascular autonomic dysfunction in MS

Cardiovascular autonomic dysfunction has been reported to reach a prevalence of 10 to 50 % in MS patients (Merkelbach et al., 2006). Loss of sympathetic and parasympathetic control in the cardiovascular system has been reported, suggesting that demyelinating plaques and inflammatory lesions may compromise vasomotor centers in the brainstem or interfere with descending fibers of the autonomic nervous system in the spinal cord (Acevedo et al., 2000; Vita et al., 1993). In 1993, Vita and co-workers correlated cardiovascular dysfunction with demyelinating lesions, demonstrating anatomical and clinical concordance through MRI (Acevedo et al., 2000; Vita et al., 1993).

Alteration of the cardiovascular control in MS patients may result in orthostatic intolerance reflecting altered sympathetic and parasympathetic outflow to the cardiovascular system (Anlar et al., 1992; Linden et al., 1995). Dysfunction of the sympathetic nervous system has been suggested to be responsible for compromised immunological function, and hypersensitivity and up-regulated beta-adrenergic receptors of immune cells have been demonstrated in progressive MS (Karaszewski et al., 1990; Linden et al., 1995).

Diagnosis of cardiovascular autonomic dysfunction is based upon a battery of different autonomic tests, such as blood pressure and heart rate responses to postural changes, the Valsalva maneuver and the heart rate variability during metronomic deep breathing (Merkelbach et al., 2006). However, the pattern of pathological findings is inconsistent, suggesting a variability of predominantly sympathetic involvement in some patients and predominantly parasympathetic pathology in others (Merkelbach et al., 2006).

To better determine the clinical and prognostic value of parameters of cardiovascular autonomic dysfunction, we will assess the cross-sectional prevalence and severity at study onset as well as the prospective course of cardiovascular autonomic dysfunction in MS patients, and we will correlate results of a battery of cardiovascular autonomic tests (for details, see below) with scores of clinical MS severity at study onset and during the prospective 36 months follow-up of our predefined group of MS patients with the above and below mentioned enrollment criteria.

5) Bladder dysfunction in MS

The pathophysiology of autonomic dysfunction is very likely based on interference of demyelinating and inflammatory lesions with central autonomic network structures, especially in the insular region, anterior cingulum, prefrontal and ventromedial cortices, the amygdala, paraventricular hypothalamus and medulla oblongata and interfere with descending autonomic pathways across the brainstem and spinal cord (Vita et al., 1993).

Urinary tract infections caused by bladder dysfunction are a common cause for deterioration in clinical status, secondary urinary tract inflammation, and even death due to secondary complications. Thus, autonomic dysfunctions have important impact on the extent of disability and confinement concerning daily activities of MS-patients.

Voiding dysfunction is a common feature in MS-patients that leads to severe impairment of the quality of life and to social isolation. The incidence of voiding dysfunction varies from 10 to 97% (Araki et al., 2002).

Detrusor-hyporeflexia and detrusor-sphincter-dysynergia may be indicative for pontine or spinal lesions in MS-patients (Araki et al., 2003). A disconnection of the neuronal pathways between pons and the sacral spine can cause reduced sphincter relaxation during detrusor contraction which in turn may yield incomplete bladder emptying and dilatation of the upper urinary pathways with subsequent urinary tract inflammation (Fowler et al., 2009) and chronic renal failure (Fowler et al., 2009).

Consequently, we will monitor urine status and signs of urinary tract infections with dip stick assessments, post-void residual bladder volume with bladder ultrasonography (Kragt et al., 2004) and voiding dysfunction with non-invasive uroflowmetry.
To better determine the clinical and prognostic value of these parameters of autonomic bladder dysfunction and inflammation, we will assess the cross-sectional prevalence and severity at study onset as well as the prospective course of autonomic bladder dysfunction and inflammation in MS patients, and we will correlate results with scores of clinical MS severity at study onset and during a 36 months follow-up period in our predefined group of MS patients with the above and below mentioned enrollment criteria.

6) Sexual dysfunction in MS

Data regarding sexual dysfunction in MS-patients are limited. Previous studies report frequencies of erectile dysfunction as high as 60%, of ejaculatory dysfunction or orgasmic dysfunction as high as 50% and reduced sexual desire at 40% (Lundberg, 1981).
Moreover, most female MS-patients report about various sexual disorders (Kirkeby et al., 1988; Lundberg, 1981).
Depending on the localization and number of MS-lesions, there may be different subtypes of sexual dysfunction.

In patients with multiple sclerosis, impairment of the spinal cord has been identified as major cause of erectile dysfunction (Betts et al., 1994; Zivadinov et al., 1999) . Some uncontrolled surveys using postal questionnaires report erectile dysfunction in 50 to 75 % of male MS patients (Bakke et al., 1996; Zorzon et al., 1999). Studies of controlled populations also showed a high incidence of erectile dysfunction among MS-patients (Lundberg, 1981; Zorzon et al., 1999). At the beginning of the disease, there may be partial sexual deficits resulting in variable severity of erectile dysfunction with preserved nocturnal and morning erections (Betts et al., 1994).

Depending on the severity of neurological deficits, total loss of sexual function can occur (Betts et al., 1994). In addition to structural lesions, fatigue, depression, spasticity or concerns about incontinence may lead to sexual impairment. Furthermore, prolonged duration of erection can decrease the ability of orgasm and ejaculation.

Female MS-patients also report increased prevalence of sexual dysfunction associated with the severity of physical disability (Kirkeby et al., 1988; Lundberg, 1981). Based on a large uncontrolled questionnaire, *Lilius et al.* report loss of orgasm in 33%, loss of libido in 27%, and increase of spasticity during sexual intercourse in 12% of female MS patients (Lilius et al., 1976).

In a case-controlled study, Zorzon et al. found similar numbers and additionally reported decreased vaginal lubrication in 36% of the patients (Zorzon et al., 1999). Loss of orgasmic function may be the most common cause for female MS patients to seek treatment (Dasgupta et al., 2004). 62% of women suffering from advanced MS describe sensory deficits of the genital region (Hulter and Lundberg, 1995).
To better determine the clinical and prognostic value of sexual dysfunction in MS patients, we will assess the cross-sectional prevalence and severity at study onset as well as the prospective course of sexual dysfunction in MS patients, using scores of sexual dysfunction questionnaires, and we will correlate results with clinical MS severity at study onset and during a 36 months follow-up period in our predefined group of MS patients with the above and below mentioned enrollment criteria.

III) Rationale

Autonomic nervous system dysfunction has been frequently described in Multiple Sclerosis (MS) patients (Merkelbach et al., 2006) and may significantly affect quality of life of MS patients (Acevedo et al., 2000; Drory et al., 1995; Flachenecker et al., 1999; Linden et al., 1995; Nasseri et al., 1998; Vita et al., 1993).
Dysfunction may include bladder, bowel and sexual function, cardiovascular, sudomotor and pupillary function (Merkelbach et al., 2006). However, reports are heterogeneous (Acevedo et al., 2000; Drory et al., 1995; Flachenecker et al., 1999; Linden et al., 1995; Nasseri et al., 1998; Vita et al., 1993), mainly reflect function of singular organ systems (Acevedo et al., 2000; Drory et al., 1995; Flachenecker et al., 1999; Linden et al., 1995; Nasseri et al., 1998; Vita et al., 1993) at varying MS stages, and studies are mostly cross-sectional but not prospective (Acevedo et al., 2000; Drory et al., 1995; Flachenecker et al., 1999; Linden et al., 1995; Nasseri et al., 1998; Vita et al., 1993).

In common diseases such as diabetes mellitus, autonomic dysfunction is known to reduce quality of life and even life expectancy (Ewing et al., 1980; Low and Hilz, 2008). In diabetes, which is the most frequent etiology of autonomic dysfunction (Low and Hilz, 2008), occurrence of cardiac autonomic dysfunction is associated with significantly increased mortality rates (Ewing et al., 1980; Ewing and Clarke, 1987). Ewing and co-workers even reported a mortality rate of 44% at 2.5 years and 56% at 5 years in diabetic patients with symptomatic autonomic neuropathy (Ewing et al., 1980).
For example, impairment of the baroreflex is associated with a deterioration of prognosis in many diseases (Lanfranchi and Somers, 2002; Ormezzano et al., 2008; Parati et al., 2001; Sykora et al., 2009). The reflex arch depends on central autonomic network interaction and on modulation of sympathetic and parasympathetic outflow towards heart and vasculature (Eckberg and Sleight, 1992; Hilz et al., 2011a; Hilz et al., 2010). Deterioration of the baroreflex sensitivity, i.e. the degree of heart rate adjustment to changes in blood pressure and vice versa (Eckberg and Sleight, 1992; Hilz et al., 2011a; Hilz et al., 2010), correlates with increased risk of cardiovascular complications and deteriorating prognosis, e.g. in arterial hypertension, heart failure, myocardial infarction, renal failure, or stroke (Lanfranchi and Somers, 2002; Ormezzano et al., 2008; Parati et al., 2001; Sykora et al., 2009), while improvement of baroreflex sensitivity, correlates with improved prognosis (Chan et al., 2005; Chan et al., 2008; Parati et al., 2001).
So far, autonomic testing is not common standard of the routine assessment of MS patients (de Seze et al., 2001b), very likely due to insufficient access of centers focusing on MS research and therapy to adequate technology, and due to inadequate experience with testing procedures (Hilz, 2002; Hilz and Dutsch, 2006).
However, autonomic parameters may serve as indices of disease severity and may augment and stratify prognostic evaluation of patients (Lanfranchi and Somers, 2002; Ormezzano et al., 2008; Parati et al., 2001; Sykora et al., 2009), and might therefore be a useful addition to the clinical assessment of MS patients.

The aim of this study is to determine the prevalence and severity of autonomic pupillary, cardiovascular, and urogenital dysfunction, in a cross-sectional study of 100 patients with relapsing remittent forms of Multiple Sclerosis (MS), and to evaluate whether parameters of autonomic dysfunction correlate with clinical MS severity. The value of autonomic parameters for estimating disease prognosis shall be determined in the same patient population in a prospective 36 months follow-up analysis of indices reflecting clinical severity and autonomic dysfunction.

IV) Ethics approval

The protocol and the proposed informed consent form will be reviewed and approved by the Ethics Committee of the University of Erlangen-Nuremberg, Erlangen, Germany.

A signed and dated statement that the protocol and informed consent have been approved by the Ethics Committee of the University of Erlangen-Nuremberg, Erlangen, Germany.

V) Patient Population and Selection

This study will include patients with relapsing-remittent MS at a rather well defined stage of disease.
We shall include only patients who have already undergone disease modifying therapy with glatiramer acetate or interferone beta treatment but still experience disease progression and MS relapses.
We will ask patients for their participation in our evaluation who have a high disease activity under treatment with baseline therapies, i.e. patients who had at least one relapse in the previous year under therapy and who display at least 9 T2-hyperintense lesions or at least one gadolinium enhancing lesion in a cranial magnetic resonance imaging (MRI) study despite β-interferon or glatiramer acetate therapy. Additionally, untreated patients with severe, rapidly progressing, relapsing-remitting MS will be included, i.e. patients who had two or more relapses with progression of disability in the previous year and who display one or more gadolinium enhancing lesions ,or a significant increase of T2-hyperintense lesions compared to the previous cranial MRI. Typically, these are MS patients who are eligible for escalation treatment with Fingolimod or natalizumab.Therefore, we will enrol patients in our study who have been offered a disease modifying therapy with Fingolimod independently from our study and prior to our request for participation in the study evaluating correlations between MS course and severity and ANS dysfunction.
The decision of the primary neurologist to put a patient on Fingolimod treatment will be made independently from and prior to our asking the patient whether he or she would be willing to participate in the comparison of ANS dysfunction with clinical MS severity.
We will enrol 100 MS patients aged 20 to 75 years, after their MS treating neurologist and the patient have agreed on the Fingolimod therapy and after the patient subsequently was informed about our study and has then given written informed consent to participate in our study.

Additional Inclusion Criteria

- Patients, or a willing and able legal representative of the patient, must provide written informed consent.
- Patients with normal orientation to person and personal situation, place, time, schedule, and temporal continuity. If there are any reasonable doubt regarding normal orientation, patients will not be included in the study.
- Patients, who are fully aware of the purpose of the study and the study procedures. Therefore, the study will be first explained in detail and then patients will be asked to repeat what they have understood. Only patients who fully understood the purpose of the study and the study procedures will be considered able to sign the consent forms.
- Abstinence from smoking, eating or drinking products containing caffeine for 18 hours prior to the study.

B Exclusion Criteria

Patients will be excluded from this study if they do not meet the specific inclusion criteria, or if:

- Patients below 20 and above 75 years;
- Patients with pre-existing diseases known to affect the autonomic nervous system.

VI) Investigational Plan and Methods

To minimize the confounder of heterogeneous duration of disease and disease modifying therapy, and to assure that the time course of our correlations is comparable among the enrolled patients, we will determine ANS function and clinical diseases severity in all patients fulfilling enrolment criteria prior to the first Fingolimod treatment, within 24 hours after the first therapy, after 6 months ± 2 weeks upon enrolment, after 12 months ± 2 weeks upon enrolment, after 24 months ± 2 weeks upon enrolment, after 36 months ± 2 weeks upon enrolment.

1) Enrollment of patients

The recruitment phase is estimated to take up to 18 months. Given individual follow-up examination over 36 months, the entire study duration will be approximately 4.5 years. The first patient is expected to be enrolled in the first half of 2012, depending on the approval of the study protocol by the Ethics Committee of the University of Erlangen-Nuremberg.

2) Cross sectional and 36 months prospective evaluation of disease severity

To monitor clinical parameters of MS during a 36 months follow up study, we will perform the *Expanded Disability Status Scale* (EDSS), assessing the degree of neurological impairment and the *Multiple Sclerosis Functional Composite* (MSFS), assessing leg, arm and cognitive function.

2.1) Expanded Disability Status Scale (EDSS):

The Expanded Disability Status Scale (EDSS) is a well standardized method of evaluating the degree of neurological impairment in MS within 8 Functional Systems (FS). The FS comprise the Pyramidal-, Cerebellar-, Brain Stem-, Sensory-, Bowel & Bladder-, Visual-, Cerebral system, and other functions (Kurtzke, 1983).
We will determine the degree of impairment by means of a neurological examination.
Each FS will be evaluated separately and rated from 0 to 6 (0 = normal to 5 or 6 = maximal impairment).
According to the results of each FS, the patient's ability to walk and the limitations in daily life, we will rate the degree of neurological impairment on a scale ranging from 0.0 to 10.0 (Kurtzke, 1983).

2.2) Multiple Sclerosis Functional Composite (MSFC):

The MSFC is a simple test to evaluate neurological function, i.e. lower extremity, upper extremity and cognitive function. The test consists of 3 objective quantitative tests (Cutter et al., 1999).
The *Timed 25-Foot Walk* measures functional capacity of the legs. It will be the first component of the MSFC administered at each visit. The patient will be directed to one end of a clearly marked 25-foot course (=7.6 meters) and instructed to walk 25 feet as quickly as possible, but safely. The patient will be accompanied by a person, who measures the time. The test will be performed twice and the mean value of both test results will be calculated (Cutter et al., 1999).

The *9-Hole Peg Test* (9-HPT) is a quantitative measure of upper extremity (arm and hand) function. The 9-HPT will be the second component of the MSFC to be administered. Two consecutive trials of the dominant hand will be followed by two consecutive trials of the non-dominant hand, using a standardized 9-HPT apparatus.
The patient will be instructed to pick up pegs one at a time, using one hand only, and put them into the holes as quickly as possible in any order until all the holes are filled. Then, the patient will have to remove the pegs one at a time and return them to the container as quickly as possible. We will determine the times needed for each test and calculate the average value (Cutter et al., 1999).

The *Paced Auditory Serial Addition Test* (PASAT) is a measure of cognitive function specifically assessing auditory information processing speed and flexibility, as well as calculation ability.
The PASAT will be the last measure of the MSFC that is administered at each visit.
On a tape (or CD) the patient will be presented a series of 60 single digit numbers that will be presented at the rate of one every 3 seconds. The patient will be instructed to listen for the first two numbers, add them up, and tell the answer. Then, the patient will have to continue to add the next number to each preceding one. The test score is the number of correct sums given (out of 60 possible) in each trial (Cutter et al., 1999).

We will calculate Z-scores for each component of the MSFC and average them to create an overall composite score known as the MSFC score (Cutter et al., 1999).

3) Infrared light reflex pupillography

Changes in pupillary light reflex responses reflect improved or deteriorated central and cranial autonomic function. Their possible association with MS progression or improvement (Frohman et al., 2005; Jakobsen, 1990) will be tested by correlating parameters of pupillary function with parameters of clinical MS severity.

Therefore, we will apply infrared light to illuminate the iris and pupil continuously. Due to different degrees of absorption, the iris and pupil reflect different quantities of the infrared light. We use a pupillograph that allows to record spontaneous fluctuations of the pupillary diameter over several minutes as well as to automatically analyze the pupillary responses to standardized light stimuli (CIP9.08™, AMTech, Weinheim, Germany). A sensor of the pupillograph, a horizontal charge coupled device (CCD) line, scans the reflection of the emitted infrared light and allows identification of the instantaneously changing margin between the iris and the pupil as a breaking point in the intensity of reflected light (Hilz, 2002). The momentary diameter of the pupil is automatically marked on a video-screen by horizontal bars (Hilz, 2002). The changes of pupillary diameter are recorded with a sampling rate of up to 250 Hz and stored on a personal computer after transmission via an analogue output of the pupillograph (Hilz, 2002).

We evaluate modulation of the pupillary diameter after a period of 45 minutes of dark-adaptation to a background illumination of 1.25 foot-candles (13.46 lux). Subjects are instructed to look at a target point mounted at a distance of at least 3 m to prevent the pupillary near response or accommodation adjustments (Blumen, 1995; Smith, 1993).

Pupillary light reflexes can be elicited by a standardized stimulus e.g. from a light emitting diode (LED) with a brightness of 10^4 cd and a flash duration of 200 milliseconds (Hilz, 2002). Consecutive changes of pupillary diameter are recorded for 2 seconds (Hilz, 2002). **Light reflex responses will be assessed for each eye by averaging four artifact-free responses to light stimulation.**

Specifically, we will assess the following static and dynamic parameters: pupil size, light reflex amplitude and relative amplitude, i.e. the percentage change related to the initial diameter, the constriction velocity and the early and late redilatation velocities. The latency and velocity of constriction and dilatation depend on the constriction amplitude which again is influenced by the resting diameter of the pupil. Therefore, latencies increase and velocities of constriction or dilatation decrease with decreasing amplitudes (Smith, 1993). The latency and constriction velocity reflect parasympathetic activity (Smith, 1993). Redilatation shows an early rapid recovery and a secondary more gradual return to the resting diameter (Hilz, 2002). The late redilatation velocity is considered to primarily reflect sympathetic activity (Smith, 1993). The time to three-quarter dilatation reflects sympathetic activity (Hilz, 2002). A delay of this redilatation is a sensitive index of peripheral sympathetic dysfunction (Smith, 1993). In darkness, the resting pupillary

diameter is highly reproducible with coefficients of variation averaging 3 % (Hilz, 2002). The pupil size depends on the degree of inhibition of parasympathetic outflow and on the amount of peripheral sympathetic tone. Decrease in sympathetic tone and changes in supranuclear inhibition contribute to the decrease of pupil size seen in adults with increasing age (Smith, 1993; Smith and Dewhirst, 1986). In diabetic patients, small dark adapted pupils indicate sympathetic deficit of autonomic activity (Hilz, 2002). The absolute and relative constriction amplitudes and the constriction velocity predominantly depend on parasympathetic activity (Heller et al., 1990; Piha and Halonen, 1994; Smith, 1993). The reflex amplitude is reduced in subjects with a small resting diameter due to age or sympathetic impairment (Smith, 1993; Smith and Dewhirst, 1986).

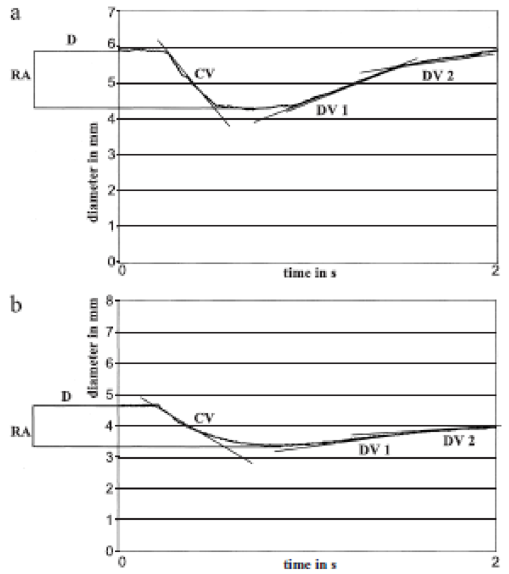

Infrared light reflex pupillography: (left: examination; right: changes in pupillary diameter in response to 104 cd light stimulation for 0.2 seconds and after dark-adaptation to 13.43 lux background illumination; brisk and pronounced light reflex curve in a 41 year-old control person (upper graph a), and attenuated, flattened pupillary light reflex curve in a 43 year-old diabetic patient (lower graph b).
(RA = reflex amplitude; D = diameter; CV = constriction velocity; DV 1 = early re-dilation velocity; DV 2 = late re-dilation velocity).
(adapted from: (Dutsch et al., 2004))

4) Assessment of disturbed saccadic eye movements by Video-Nystagmography

Eye movement disorders in MS patients are predictive of severity of disease and handicap (Derwenskus et al., 2005; Serra et al., 2003).

To assess dysfunction of saccadic eye movements, a common neuro-ophthalmologic disorder in MS (Eggenberger, 1996; Frohman et al., 2005; Rougier and Tilikete, 2008), we will record saccadic eye movements using a Video-Nystagmograph (HOMOTH VNG4000) with a combination mask for partial free-sight- and dark measurement with composite adaptation to a PCI frame grabber card, measuring with 2 channel real time recording and artefact suppression horizontal. The signal resolution is defined at 0,1 degree at 704 x 288 Pixel with a signal rate of 50 Hz. The camera is using infrared light at 950 nm (limited after DIN EN 60825-1).

The experiment will take place in a dimly illuminated room. Subjects will be seated and facing a horizontally arranged arc perimeter of 90° in radius, positioned at eye level. The distance between the centre of the perimeter and the subjects' eyes will be 57 cm (Natale et al., 2007). One green LED will serve as fixation point and will be placed at the centre of the perimeter which will be aligned with the subject's body midline. Several red LEDs will be placed from 2.5° up to 30° to the right and to the left of fixation, 2.5° apart from each other, creating an array of visible LEDs among which only those located at ±30° will be lit, whereas the others will never be used as a target in the experiment (Natale et al., 2007). The arrangement of equally spaced target and non-lit LEDs has the purpose of visually compensating for the higher density of target LEDs within the first 10° as compared to other field sectors and of minimising the possibility of eccentric fixation on a specific target position. Moreover, the presence of location markers, that is, the array of visible LEDs in our case, has been shown to favour in normal subjects an eccentricity-related increase in saccadic latency, which, on the contrary, is not present in an unstructured visual field (Natale et al., 2007). 30 horizontal centrifugal saccades in either direction will be recorded for ±30° amplitudes. Comparison of independent calibrations at a 30-degree angle before and after all recordings will be used to control against artefacts.

From Video-Nystagmograph recordings, we will calculate maximal saccade velocity.

5) Cardiovascular autonomic dysfunction

Cardiovascular autonomic dysfunction has been reported to reach a prevalence of 10 to 50 % in MS patients (Merkelbach et al., 2006). Loss of sympathetic and parasympathetic control in the cardiovascular system has been reported, suggesting that demyelinating plaques may damage vasomotor centers in the brainstem or interfere with descending fibers of the autonomic nervous system in the spinal cord (Acevedo et al., 2000; Vita et al., 1993).
A **questionnaire** assessing autonomic dysfunction will be used for baseline and follow-up examinations (see below).
Changes in scores of autonomic function and parameters of cardiovascular autonomic function reflect improved or deteriorated autonomic function. The possible association of such changes with MS progression or improvement (Frohman et al., 2005; Jakobsen, 1990) will be tested by correlating autonomic scores and parameters of parameters of cardiovascular autonomic function with parameters of clinical MS severity.

5.1) Monitoring of cardiovascular biosignals

During the assessment of cardiovascular autonomic function, we will continuously monitor the following parameters:

- Heart rate, assessed as RR interval (RRI), will be recorded by an electrocardiogram (ECG). RRI signals will be monitored using conventional superficial disc electrodes attached to the area under the right and left clavicle and the right and left iliac crest. Signals will be sampled using a Colin Pilot monitor (Colin Medical Instruments, San Antonio, Texas) (Hilz, 2002).
- Beat-to-beat blood pressure (BP) will be measured continuously and non-invasively by means of applanation tonometry (Colin Pilot; Colin Medical Instruments, San Antonio, TX). A bracelet-like sensor is attached to the left wrist using a velcro-strap. The tonometer consists of the sensor, an array of 31 equally spaced piezoresistive pressure transducers, an automated positioning system, and uses signal conditioning and initial calibration by oscillometric cuff measurement of brachial artery blood pressure (Hilz, 2002). The sensor measures radial artery blood pressure by tonometric applanation technique. The tonometer is calibrated in 5-10 min intervals by means of a conventional blood pressure cuff attached to the upper left arm.
  Alternatively, blood pressure will be recorded continuously from the right hand using finger pulse photoplethysmography (Portapress; TPD Biomedical Instrumentation, Amsterdam, The Netherlands).
- Thoracic and abdominal respiration will be monitored after calibration using respiratory belts based on piezoelectric principles. One belt will be attached to the lower thorax, the second belt will be attached around the mid-abdomen, at the points of maximal respiratory excursion (Hilz, 2002).

- Capillary blood flow of the right and left index finger pulp as well as the right and left second toe will be continuously monitored by means of a Periflux four channel laserflow Doppler (Perimed, Stockholm, Sweden) (Hilz, 2002).
- Skin-conductance serves as a measure of sympathetic outflow (Hilz, 2002) and can be monitored at the left middle finger and the left thenar using two superficial electrodes. By inducing continuous voltage of 0.5 V between the two electrodes, the electric conductivity of the skin will be measured as current circulating between the two electrodes. The skin-conductance G (Micro-Siemens [µS] or [µmho]) can be calculated as ratio between current I and voltage U (Dawson M.E., 2000)

Results of the above tests at baseline and during the follow-up examinations will be correlated with parameters of clinical MS severity.

5.2) Data processing

All bio-signals will be sampled, digitized and displayed on a personal computer and a custom designed data acquisition and analysis system (SUEmpathy™, SUESS Medizin-Technik GmbH, Aue, Germany) and stored for off-line analysis.

We will determine mean values and standard deviation of all bio-signals, e.g. of RR-interval (RRI), blood pressure, superficial skin blood flow, transcutaneous oxygen saturation and skin conductance. We will calculate underlying sympathetic and parasympathetic cardiovascular modulation (Rudiger et al., 1999).

Assuring stable baseline signals:

To assure stability of values before autonomic testing, we will calculate the baseline or resting values of two 180 sec recording segments. To verify stability of signals at baseline, we will compare mean values and standard deviations of all bio-signals and parameters averaged during each of the two 180 second recordings (Bernardi et al., 1995b). Stationarity will be assumed if the mean values of the signals from the first and second 3-minute epoch will not differ by more than 10% and if there will not be a statistical difference between the standard deviations (Bernardi et al., 1995b).

5.3) Autonomic bio-signal variability at rest

Impairment of sympathetic and parasympathetic cardiovascular control is likely to occur in MS patients and suggests that MS pathology such as demyelination or inflammation interferes with structures of autonomic control in supratentorial as well as brainstem and spinal cord areas (Acevedo et al., 2000).

Even under resting conditions, healthy persons show a variability of heart rate, blood pressure, and skin conductance show variability with signal oscillations at low frequencies (Berger et al., 1986).
During the resting period, we will record 5-min time series of heart rate monitored as RRIs in order to analyze heart rate variability (HRV) by calculating the mean of the RRI, the RRI standard deviation (RR-SD), and the root mean square of successive differences between adjacent RRIs (RMSSD) (1996). RMSSD reflects mainly parasympathetic modulation of HR, whereas RR-SD reflects sympathetic and parasympathetic HR modulation (1996).

In addition, we will assess the contribution of the sympathetic and parasympathetic systems to heart rate and BP modulation under resting conditions as well as during autonomic challenge maneuvers. We will evaluate RR interval, and BP variability by means of power spectral analysis, using the so-called trigonometric regressive spectral analysis algorithm (TRS) (Rudiger et al., 1999).

We will identify peaks of oscillations in the low frequency (LF: 0.04–0.14 Hz) and high frequency (HF: 0.15–0.50 Hz) ranges. LF oscillations of RRI at rest are considered to be mediated by combined sympathetic and parasympathetic activity, whereas there is a predominance of sympathetic activity during stressful conditions (1996; Saul et al., 1989). HF oscillations in RRI are associated with respiratory sinus arrhythmia and reflect parasympathetic activity (1996), whereas fluctuations of the BP signal in the HF range are primarily a mechanical consequence of respiration-induced fluctuations in venous return (1996; Saul et al., 1989).

Since patients with MS might show rapid changes in various bio-signals with pronounced signal fluctuations and lack of adequate bio-signal stationarity, short recording epochs must be analyzed. Standard algorithms such as Fast Fourier Transformation are not well suited for this approach (Hilz et al., 2011b). Instead, we will apply algorithms that provide better time resolution and evaluation of short term bio-signal changes, such as the so-called trigonometric regressive spectral analysis (TRS) algorithm that yields a high grade of accuracy even for 30s intervals (Rudiger et al., 1999), or autoregressive algorithms as described e.g. by Bernardi et al. (Bernardi et al., 1995a).

We will assess sympathetic and parasympathetic influences on the variability of bio-signals such as RRI and BP by using the TRS or autoregressive algorithms and quantifying the LF and HF components of these signals. The magnitude of the two components will be determined as the

integral under the power spectral density curves of RRI (ms^2/Hz) and BP ($mmHg^2/Hz$) for the two frequency bands, and will be expressed as LF and HF powers of RRI (ms^2) and BP ($mmHg^2$).

We will normalize the powers of RRI oscillations (LFnu, HFnu) as percentage values by dividing the LF or HF power by the sum of the LF and HF powers and multiplying by 100 (1996). Because the LF and HF powers of the RRI and BP signals might show a skewed distribution, we might have to transform the powers into natural logarithms (Hilz et al., 2006).

5.4) Heart rate and blood pressure responses and spectral powers of autonomic modulation during active standing

For testing heart rate and blood pressure responses to postural change, we will perform a postural blood pressure test, the so-called Schellong test (Hilz, 2002). After a resting phase of 10 min in the supine position, the participant will be asked to stand up and to remain standing for further 10 minutes. During the test, heart rate and blood pressure will be continuously monitored.

The arm wearing the blood pressure cuff should rest comfortably at heart level and will be held in place using a Velcro-strap. In healthy persons, standing up results in minimal changes of blood pressure (Mathias and Bannister, 1993). In patients with autonomic disorders, there may be a significant drop in mean blood pressure following standing (Mathias and Bannister, 1993). Maintaining blood pressure in the orthostatic position depends on peripheral vasoconstriction and heart rate increase which are mediated by activation of the sympathetic nervous system and by withdrawal of parasympathetic cardiac activity (Hilz, 2002; Mathias and Bannister, 1993).

The Schellong test will be considered positive when the systolic pressure decreases by more than 20 mm Hg or drops below 90 mm Hg (Hilz, 2002). In patients, who cannot perform active standing, we will evaluate cardiovascular responses to passive orthostatic challenge by means of 90° head-up tilt (Hilz, 2002).
The active or passive orthostatic challenge will be discontinued if participants show signs and symptoms of orthostatic hypotension or presyncope, such as nausea, dizziness, beginning headache, blurred vision or other complaints of discomfort, or if there is a drop in blood pressure by more than 30 mmHg systolic or more than 20 mmHg diastolic, or an increase in heart rate by more than 35 beats per minute or above 135 beats per minute.

5.5) Assessment of baroreflex sensitivity

We will assess baroreflex sensitivity (BRS) at resting conditions and during active or passive standing using various algorithms including the so called alpha-index (Hilz et al., 2011a) or the algorithm described by Parati et al. that differentiates between sequences of increases in blood pressure and RRI values as well as sequences of decreases in blood pressure and RRI values (Parati et al., 1988).

Beat-to-beat analysis of the continuous relationship between spontaneous fluctuations in heart rate and blood pressure shows sequences of continuous beats in which systolic arterial pressure increases and heart rate decreases, or vice versa (Bertinieri et al., 1985; Legramante et al., 1999; Parati et al., 1988). Baroreflex sensitivity can be calculated as the slope of the regression between spontaneously occurring ramps of blood pressure increase or decrease and subsequent RR interval changes (Fritsch et al., 1986; Parati et al., 1988).

As an alternative measure of BRS we might also use the method described by Robbe et al. (Robbe et al., 1987) who demonstrated that BRS can be assessed by analyzing the relationship between spontaneous sympathetically mediated fluctuations of the BP signal, reflecting the input activity of the baroreflex, and corresponding RRI fluctuations, reflecting the reflex output activity (Robbe et al., 1987). The amplification between the input and output of the reflex is an index of BRS (Robbe et al., 1987). Mathematically, this amplification equals the gain of the transfer function between the oscillations of BP and RRI in the LF range, provided there is sufficient coherence (> 0.5), i.e. a stable relationship between both bio-signals (Hilz, 2002; Robbe et al., 1987).

The coherence between BP and RRI signal oscillations might span from 0 (i.e. no association) to 1 (i.e. maximal association). Two signals will be considered to have a stable phase relation for a given frequency of oscillation if coherence is above 0.5 (Bernardi et al., 1995b).

In addition, the LF gain between systolic BP and RRI oscillations may be obtained by calculating the α-index, i.e. the square root of the ratio of LF-power of RR-interval to LF-power of systolic BP (ms/mmHg). The α-index will be calculated only if there is significant coherence (> 0·5) between both oscillations, i.e. the two signals have a stable phase relation (Hilz, 2002; Pitzalis et al., 1998).

5.6) Blood pressure and heart rate responses during the Valsalva maneuver

In MS patients, we expect to see altered responses to the Valsalva maneuver as the baroreflex mediated reflex bradycardia after release of the expiratory strain is modulated by central areas of autonomic control (Hilz, 2002).
Central areas of autonomic control, especially the cardiovascular autonomic nuclei (nucleus tractus solitarii and hypothalamic nuclei) are located in the periventricular region of the fourth ventricle; which is a common site of lesions in MS (Oppenheimer, 1976).

Heart rate will be tested in response to two reproducible Valsalva maneuvers. The Valsalva maneuver tests the afferent, central, and efferent sympathetic and parasympathetic baroreflex pathways (Hilz, 2002). The Valsalva maneuver will not be performed in patients with retinopathy, glaucoma, cerebral aneurysms, dissections or increased intracranial pressure.

The Valsalva maneuver is usually standardized by asking the subject to blow into a mouthpiece to a pressure of 40 mm Hg measured on an aneroid manometer, and continue blowing for 15 s while the HR is continuously recorded (Ewing, 1992; Hilz, 2002). The measure of the HR response is the Valsalva ratio which is defined as the ratio between the highest heart rate during and the lowest heart rate within the first 20 s after the Valsalva maneuvers (Ewing, 1992; Hilz, 2002). The value for each subject will be calculated for each of the two Valsalva maneuvers.

The response to Valsalva maneuver includes four phases. In normal subjects, the sudden increase of intrathoracic pressure results in a brief rise in blood pressure due to mechanical factors and in a brief fall of HR due to parasympathetic activation (phase I).
The ongoing strain (phase II) reduces the venous return to the heart which results in reduction of ventricular dimensions, left ventricular stroke volume and cardiac output. This triggers reflex tachycardia and vasoconstriction. The tachycardia during phase II is induced by a prominent early component of inhibition of cardiovagal output and a late component, the increased sympathetic output. In normal subjects, phase II consists of an early fall of arterial blood pressure and subsequent partial recovery. The recovery during the late phase II is a result of the progressive increase in total peripheral resistance due to increased sympathetic activity.
In phase III the fall of arterial blood pressure after the release of intrathoracic pressure reflects mechanical factors. The HR shows a reflex increase for usually 3-4 beats.
The last phase of the normal response to the Valsalva maneuver is a rebound overshoot of blood pressure due to the persistent vasoconstriction of the arteriolar bed and to the increased cardiac output that occurs upon release of the forced inspiration (Ewing, 1992, 1993; Hilz, 2002).
Stimulation of baroreceptors by the increase in blood pressure in phase IV induces reflex bradycardia due to increased parasympathetic activity (Ewing, 1992, 1993; Hilz, 2002).
The arterial blood pressure changes in the second and in the last phase can be used as tests of sympathetic function (Hilz, 2002). The reflex bradycardia in phase IV can be used as a test of parasympathetic function (Hilz, 2002).

In MS patients with autonomic dysfunction, we might see a characteristic blood pressure pattern that typically shows no overshoot after release of the expiratory strain because of compromised reflex pathways. Moreover, blood pressure is likely to show a slow and steady decline during the early and late phase II of the strain as cardiac output falls, and a gradual return to normal with no rebound blood pressure rise after release of intrathoracic pressure (Hilz, 2002). Moreover, there might be almost no change in heart rate throughout the entire test, with no apparent reflex bradycardia in phase IV (Ewing, 1992, 1993; Hilz, 2002).

Cutaneous blood flow decreases in the second phase of the maneuver due to sympathetic pathways (Ewing, 1992, 1993; Hilz, 2002).

5.7) Heart rate variation during 3-minute deep breathing

MS patients are likely to have abnormal heart rate modulation with metronomic deep breathing, i.e. altered sinus arrhythmia, as the respiratory modulation of heart rate involves multiple central and peripheral structures of the autonomic nervous system. Among the various cardiovascular autonomic tests performed in MS patients, the heart rate response during deep breathing most frequently showed abnormal results (Nasseri et al., 1998; Pentland and Ewing, 1987; Vita et al., 1993).

Respiratory sinus arrhythmia depends on the interaction of multiple peripheral and central autonomic structures and reflex arcs including outflow from the respiratory center to the medullary vagal efferent neurons, centrally mediated changes in baroreflex sensitivity, the Hering-Breuer reflex activated during inspiration by stretch receptors in the lungs and chest wall, the Bainbridge reflex, etc. (Low, 1997).

While heart rate is monitored as RRIs, the participants will be asked to breathe deeply at a frequency of 6 cycles per minute which has been shown to produce maximal HRV in healthy individuals (Hilz, 2002). The inspiration and expiration intervals will be 5s each. The heart rate, coefficient of variation, and root-mean-squared-successive-difference (RMSSD) are computed from 10 R-R intervals without artifacts. In the breathing cycle with the maximal HRV, the longest RR interval (RRmax) during expiration and the shortest RR interval (RRmin) during inspiration will be determined to assess the expiratory-inspiratory difference (E-I-difference = RRmax - RRmin) and the expiratory-inspiratory ratio (E/I ratio = RRmax/RRmin) (Hilz, 2002; Sundkvist et al., 1979; Wheeler and Watkins, 1973).

5.8) Blood pressure response to sustained handgrip test

Cardiovascular autonomic function in MS has been assessed by several authors who found abnormal results in a large number of MS patients (Anema et al., 1991; Mutani et al., 1982; Nordenbo et al., 1989; Pentland and Ewing, 1987; Senaratne et al., 1984; Sterman et al., 1985). According to Vita et al., abnormal results were seen with different autonomic tests *without* a consistent pattern, however, the most frequent abnormalities were recorded with the metronomic deep breathing test and with tests assessing sympathetic function (Vita et al., 1993). The sustained handgrip test evaluates sympathetic activation with continuous sub-maximal physical effort (Hilz, 2002).

First, the participant will be asked to press a handgrip dynamometer with full strength. Then, the handgrip should be maintained for 3-5 min at one-third of the maximum contraction strength. The subsequent increase of sympathetic activity and vasoconstriction induces a blood pressure rise that is considered to reflect sympathetic autonomic activity. The early acceleration of heart rate during the maneuver is due to a withdrawal of vagal activity, whereas the late heart-rate acceleration results from sympathetic activation. Normally, diastolic blood pressure at the end of the effort is at least 16 mmHg higher than before the maneuver. A diastolic blood pressure increase by only 10 mmHg or less is abnormal. We will assure that the patients do not perform a Valsalva maneuver during the handgrip test that is a common bias of the test results (Hilz, 2002; Hilz and Dutsch, 2006).

5.9) Classification of autonomic cardiovascular function

Based on the results from the standard heart rate and blood pressure tests, i.e. heart rate and blood pressure responses at rest, during orthostatic challenge, during metronomic breathing, the Valsalva maneuvers and the sustained handgrip test, we will consider five categories of cardiovascular involvement.

Results will be considered normal if all tests are normal or one test shows borderline results. Mild cardiovascular dysfunction will be diagnosed if one of the three heart rate tests is abnormal or if two tests yield borderline results.

We will diagnose definite autonomic cardiovascular dysfunction if two or more heart rate tests are abnormal.

We will diagnose severe autonomic cardiovascular dysfunction if two or more of the heart rate tests are abnormal and one or both of the blood pressure tests are abnormal, or if both BP tests yield borderline results (Ewing et al., 1985).

6) Assessment of bladder function

Most MS patients develop lower urinary tract symptoms and sexual dysfunction at some stage of the disease (DasGupta and Fowler, 2002; Fernandez, 2002).

There are even associations between the duration of MS and urinary tract dysfunction (Koldewijn et al., 1995). In the early stage of the disease, a low percentage of MS patients have urological complaints, sometimes associated with other neurological symptoms (Miller et al., 1965), although there may be evidence of urological dysfunction in clinically silent patients (Bemelmans et al., 1991).

During the course of the disease, most patients with MS develop urinary symptoms, women and men being equally affected. In one series, symptomatic voiding dysfunction was present in 97% of the patients, urgency and frequency in 32%, incontinence in 49%, and hesitancy-retention in 19% (Goldstein et al., 1982).

Different prevalence in various reports might be due to methodological factors or recruitment of patients from different hospital settings and at different stages of MS. Therefore, we will take the above described measures and use the above inclusion criteria to assure adequate homogeneity of disease severity and treatment stages or duration.

Changes in bladder function, including micturition urgency, detrusor hyperreflexia, detrusor-sphincter dyssynergia, detrusor hyporeflexia or secondary urinary tract infection reflect improved or deteriorated autonomic bladder function. The possible association of such changes with MS progression or improvement (Frohman et al., 2005; Jakobsen, 1990) will be tested by correlating parameters of bladder function and infection with parameters of clinical MS severity.

Micturition urgency, detrusor hyperreflexia, detrusor-sphincter dyssynergia, detrusor hyporeflexia will be assessed by questionnaires, ultrasound evaluation of post-void residual bladder volume and uroflowmetry (de Seze et al., 2007; Nakipoglu et al., 2009).

Urinary tract function and inflammation, we will be assessed by means of a questionnaire evaluating lower urinary tract dysfunction such as burning micturition, micturition urgency, and frequency bladder incontinence.

6.1) Assessment of the urine status with dip stick

To evaluate frequently occurring urinary tract infection related to autonomic bladder dysfunction, we will use a simple screening method for urinary tract infection, a dip-stick test.

Changes in dip-stick results may reflect improved or deteriorated autonomic bladder function. The possible association of such changes with MS progression or improvement will be tested by correlating dip-stick results with parameters of clinical MS severity.

Urine dip-stick analysis will determine, e.g. urine pH and presence of leukocytes or nitrite indicating bacterial inflammation, protein showing upper urinary tract dysfunction, as e.g. in glomerulonephritis, erythrocytes showing hemorrhagic inflammation by presence of nitrite. Dip-stick tests reliably diagnose urinary tract inflammation in MS patients. The tests employ chemical on a stick dipped in urine that reacts e.g. to nitrites, substances produced by many of the bacteria causing urinary tract infections. A positive result indicates urinary infection while negative testing helps avoid unnecessary antibiotic treatment (Patel et al., 2005).

6.2) Post-voiding residual urine sonography

After a patient has voided the bladder normally, we will measure the post-void residual bladder volume by ultrasound examination as described below. We will moreover ask the patients whether they usually apply special maneuvers to facilitate bladder emptying, e.g. abdominal straining, bending forward, applying manual pressure to the lower abdominal wall. If patients applied such maneuvers, the determined residual volume would not reflect the actual status of detrusor contractility.
Therefore, we will ask patients to refrain from any of these maneuvers when voiding the bladder during any of our examinations.

If MS patients are unable to completely empty their bladder, they are at increased risk of deterioration of storage dysfunction. They may perceive a sensation of incomplete bladder emptying, bladder capacity may change and is frequently reduced while residual urine volume increases. Thus, the micturition interval shortens while frequency of micturition, urinary urgency and urge incontinence and risk of bladder infection increase with increasing residual urine volume (Brady and Fowler, 2001; Stewart and Fowler, 1999).

To assess post-voiding urinary residual volume, we will perform ***bladder sonography***.
The patients will be in lying, supine position, we will place a 3.5-MHz ultrasound transducer in midline position and maximize the view in the longitudinal bladder section; then, the ultrasound probe will be rotated by 90° without changing the contact points, by changing the insonation angle upwards and downwards, we will identify the largest transverse bladder area (Amole et al., 2004). In the longitudinal as well as the transverse position, we will measure the two orthogonal

diameters of each of the two bladder sections (Amole et al., 2004). Thus, we will take the maximum longitudinal and antero-posterior diameters on the maximal longitudinal image as well as the maximum transverse diameter and height on the maximal transverse image (Amole et al., 2004). Bladder volume will be calculated according to the formula “volume = 0.6 X height X diameter X width (Byun et al., 2003). The residual urine volume will be recorded before and at each of the follow-up examinations.
Changes in residual urine volume may reflect improved or deteriorated autonomic bladder function. The possible association of such changes with MS progression or improvement will be tested by correlating residual urine volume with parameters of clinical MS severity.

6.3) Uroflowmetry

Uroflowmetry is an easily performed non-invasive method to assess voiding disorders. The patient voids into a beaker and the monitoring unit assesses voided urine volume (ml), the maximum flow rate (ml/s), average flow rate (ml/s), voiding time (sec), flow time (sec), time to maximum flow (sec) and hesitancy (sec). In healthy persons, the flow curve of voided urine per time usually has a bell-shaped curve (Brady and Fowler, 2001; Stewart and Fowler, 1999).

In MS patients, the uroflow pattern is frequently disturbed to e.g. sphincter dyssynergia, detrusor hyperactivity (Nakipoglu et al., 2009).

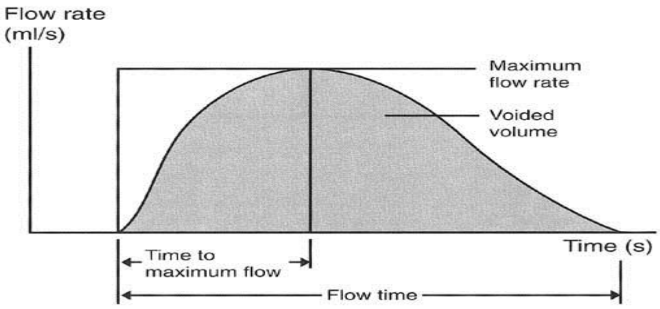

Normal Uroflow curve
(from: http://www.medcases.com/simulations/case_613/media/pix/a0001903.jpg)

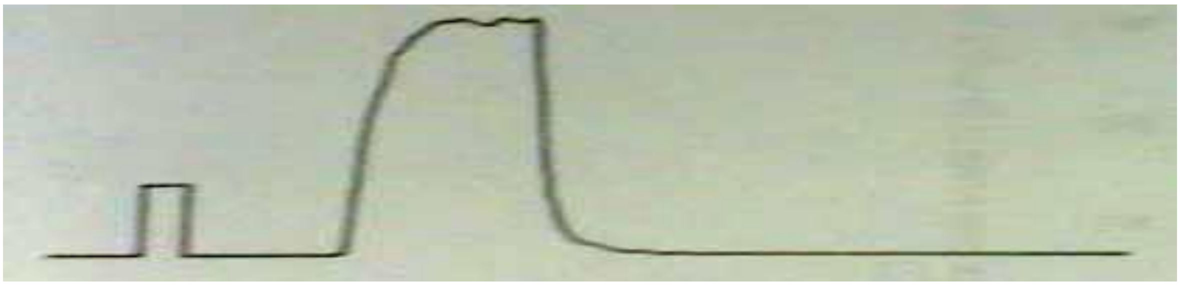

Detrusor hyperactivity with high maximum flow rate achieved within abnormally short time.
(from: http://www.medindia.net/articles/Manual-Urodynamics-print.htm)

We will assess parameters of uroflowmetry at baseline and during follow-up examinations. Changes in parameters of uroflowmetry may reflect improved or deteriorated autonomic bladder function. The possible association of such changes with MS progression or improvement will be tested by correlating the parameters of uroflowmetry with parameters of clinical MS severity.

7) Questionnaires assessing sexual dysfunction, bladder dysfunction and autonomic symptoms

Several questionnaires will be presented to the study participants.

Questionnaires are in German as the participants in this initial observational study of correlations between parameters of autonomic dysfunction and parameters of clinical MS severity speak German.
All study participants will receive questionnaires assessing various dysfunctions frequently occurring in MS and progressing with disease severity.
Changes in scores of questionnaires may reflect improved or deteriorated autonomic function. The possible association of such changes with MS progression or improvement will be tested by correlating the scores of questionnaires with parameters of clinical MS severity.

The questionnaires address the following aspects of dysfunction:

a) sexual dysfunction
b) bladder and micturition dysfunction
c) symptoms of autonomic nervous system dysfunction

The patients should fill in the questionnaires by themselves but can receive explanatory information upon request. Completing the questionnaires is estimated to take 45 minutes. After completion, all participants have the opportunity for further conversation and information regarding the issues addressed in the questionnaires

VII) Statistical analysis

For analysis of data a commercially available statistical program (SPSS™, SPSS Inc., Chicago, IL, USA) will be used.

We will test data for normal distribution by the Shapiro-Wilk test. In case of normally distributed data, we will use analysis of variance for repeated measurements (ANOVA, general linear model), with the main effect tests (e.g. Valsalva maneuver, metronomic breathing with six cycles per minute; residual post-void bladder volume etc.) as within subject factors. The suitability of the ANOVA model will be confirmed by Mauchly's Test of Sphericity. In case of violation of the sphericity assumption, the Greenhouse Geisser correction will be employed. In case of significant ANOVA results, post-hoc single comparisons will be performed using t-tests for data comparison within groups. For non-normally distributed data we will use the Kruskal-Wallis-Test to assess differences in the various parameters. In case of significant results, post-hoc single comparisons will be performed using Wilcoxon-Test.

Correlation between disease progression and severity, i.e. results of the EDSS or MSFS and bio-signals as well as autonomic parameters and BRS will be assessed with the Spearman rank correlation test for non-normally distributed data or Pearson test for normally distributed data. Significance will be set at a P- value of less than 0.05.

All tests will be performed in a quiet, temperature controlled laboratory environment. The daytime of measurement will not differ by more than six hours between the measurements.

VIII) References

1996. Task Force of the European Society of Cardiology and the North American Society of Pacing and Electrophysiology: Heart rate variability: standards of measurement, physiological interpretation, and clinical use. Circulation 93:1043-1065.

Acevedo AR, Nava C, Arriada N, Violante A, Corona T. 2000. Cardiovascular dysfunction in multiple sclerosis. Acta Neurol Scand 101:85-88.

Amole AO, Kuranga SA, Oyejola BA. 2004. Sonographic assessment of postvoid residual urine volumes in patients with benign prostatic hyperplasia. J Natl Med Assoc 96:234-239.

Anema JR, Heijenbrok MW, Faes TJ, Heimans JJ, Lanting P, Polman CH. 1991. Cardiovascular autonomic function in multiple sclerosis. J Neurol Sci 104:129-134.

Anlar B, Karaszewski JW, Reder AT, Arnason BG. 1992. Increased muscarinic cholinergic receptor density on CD4+ lymphocytes in progressive multiple sclerosis. J Neuroimmunol 36:171-177.

Araki I, Matsui M, Ozawa K, Nishimura M, Kuno S, Saida T. 2002. Relationship between urinary symptoms and disease-related parameters in multiple sclerosis. J Neurol 249:1010-1015.

Araki I, Matsui M, Ozawa K, Takeda M, Kuno S. 2003. Relationship of bladder dysfunction to lesion site in multiple sclerosis. J Urol 169:1384-1387.

Bakke A, Myhr KM, Gronning M, Nyland H. 1996. Bladder, bowel and sexual dysfunction in patients with multiple sclerosis--a cohort study. Scand J Urol Nephrol Suppl 179:61-66.

Balcer LJ. 2001. Clinical outcome measures for research in multiple sclerosis. J Neuroophthalmol 21:296-301.

Bemelmans BL, Hommes OR, Van Kerrebroeck PE, Lemmens WA, Doesburg WH, Debruyne FM. 1991. Evidence for early lower urinary tract dysfunction in clinically silent multiple sclerosis. J Urol 145:1219-1224.

Benarroch EE. 1997a. The Central Autonomic Network. In: Low PA, editor. Clinical Autonomic Disorders, 2 ed. Philadelphia: Lippincott-Raven Publishers. p 17-23.

Benarroch EE. 1997b. Central autonomic network: functional organization and clinical correlations. Armonk, NY: Futura Publishing Company, Inc.

Berger RD, Akselrod S, Gordon D, Cohen RJ. 1986. An efficient algorithm for spectral analysis of heart rate variability. IEEE Trans Biomed Eng 33:900-904.

Bernardi L, Bianchini B, Spadacini G. 1995a. Demonstrable cardiac reinnervation after human heart transplantation by carotid baroreflex modulation of RR interval. Circulation 92:2895-2903.

Bernardi L, Bianchini B, Spadacini G, Leuzzi S, Valle F, Marchesi E, Passino C, Calciati A, Vigano M, Rinaldi M, Martinelli L, Finardi G, Sleight P. 1995b. Demonstrable cardiac reinnervation after human heart transplantation by carotid baroreflex modulation of RR interval. Circulation 92:2895-2903.

Bertinieri G, di Rienzo M, Cavallazzi A, Ferrari AU, Pedotti A, Mancia G. 1985. A new approach to analysis of the arterial baroreflex. J Hypertens Suppl 3:S79-81.

Betts CD, Jones SJ, Fowler CG, Fowler CJ. 1994. Erectile dysfunction in multiple sclerosis. Associated neurological and neurophysiological deficits, and treatment of the condition. Brain 117 (Pt 6):1303-1310.

Blumen S. 1995. Light and ciliospinal reflexes: pupil pharmacology, pupil cycle time, and pupillography. In: Korczyn A, editor. Handbook of Autonomic Nervous System Dysfunction. New York: Marcel Dekker. p 539-555.

Brady CM, Fowler CJ. 2001. Urinary incontinence and retention. In: Munsat TL, editor. Neurologic Bladder, Bowel

and Sexual Dysfunction, 1st ed. p 7-27.

Byun SS, Kim HH, Lee E, Paick JS, Kamg W, Oh SJ. 2003. Accuracy of bladder volume determinations by ultrasonography: are they accurate over entire bladder volume range? Urology 62:656-660.

Chan CT, Jain V, Picton P, Pierratos A, Floras JS. 2005. Nocturnal hemodialysis increases arterial baroreflex sensitivity and compliance and normalizes blood pressure of hypertensive patients with end-stage renal disease. Kidney Int 68:338-344.

Chan CT, Shen XS, Picton P, Floras J. 2008. Nocturnal home hemodialysis improves baroreflex effectiveness index of end-stage renal disease patients. J Hypertens 26:1795-1800.

Cutter GR, Baier ML, Rudick RA, Cookfair DL, Fischer JS, Petkau J, Syndulko K, Weinshenker BG, Antel JP, Confavreux C, Ellison GW, Lublin F, Miller AE, Rao SM, Reingold S, Thompson A, Willoughby E. 1999. Development of a multiple sclerosis functional composite as a clinical trial outcome measure. Brain 122 (Pt 5):871-882.

DasGupta R, Fowler CJ. 2002. Sexual and urological dysfunction in multiple sclerosis: better understanding and improved therapies. Curr Opin Neurol 15:271-278.

Dasgupta R, Wiseman OJ, Kanabar G, Fowler CJ, Mikol DD. 2004. Efficacy of sildenafil in the treatment of female sexual dysfunction due to multiple sclerosis. J Urol 171:1189-1193; discussion 1193.

Dawson M.E. SAM, Filion D.L. 2000. The electrodermal system. In: Cacioppo J.T. TLG, editor. Handbook of Psychophysiology, 2 ed. Cambridge: Cambridge University Press. p 200-223.

de Seze J, Arndt C, Stojkovic T, Ayachi M, Gauvrit JY, Bughin M, Saint Michel T, Pruvo JP, Hache JC, Vermersch P. 2001a. Pupillary disturbances in multiple sclerosis: correlation with MRI findings. J Neurol Sci 188:37-41.

de Seze J, Stojkovic T, Gauvrit JY, Devos D, Ayachi M, Cassim F, Saint Michel T, Pruvo JP, Guieu JD, Vermersch P. 2001b. Autonomic dysfunction in multiple sclerosis: cervical spinal cord atrophy correlates. J Neurol 248:297-303.

de Seze M, Ruffion A, Denys P, Joseph PA, Perrouin-Verbe B. 2007. The neurogenic bladder in multiple sclerosis: review of the literature and proposal of management guidelines. Mult Scler 13:915-928.

Derwenskus J, Rucker JC, Serra A, Stahl JS, Downey DL, Adams NL, Leigh RJ. 2005. Abnormal eye movements predict disability in MS: two-year follow-up. Ann N Y Acad Sci 1039:521-523.

Drory VE, Nisipeanu PF, Kroczyn AD. 1995. Tests of autonomic dysfunction in patients with multiple sclerosis. Acta Neurol Scand 92:356-360.

Druschky A, Spitzer A, Platsch G, Claus D, Feistel H, Druschky K, Hilz MJ, Neundorfer B. 1999. Cardiac sympathetic denervation in early stages of amyotrophic lateral sclerosis demonstrated by 123I-MIBG-SPECT. Acta Neurol Scand 99:308-314.

Dutsch M, Marthol H, Michelson G, Neundorfer B, Hilz MJ. 2004. Pupillography refines the diagnosis of diabetic autonomic neuropathy. J Neurol Sci 222:75-81.

Eckberg DL, Sleight P. 1992. Human Baroreflexes in Health and Disease. Oxford, New York: Oxford University Press.

Egg R, Hogl B, Glatzl S, Beer R, Berger T. 2002. Autonomic instability, as measured by pupillary unrest, is not associated with multiple sclerosis fatigue severity. Mult Scler 8:256-260.

Eggenberger E. 1996. Neuro-ophthalmology of multiple sclerosis. Curr Opin Ophthalmol 7:19-29.

Ewing DJ. 1992. Analysis of heart rate variability and other non-invasive tests with special reference to diabetes mellitus. In: Bannister R, Mathias CJ, editors. Autonomic Failure. A Textbook of Clinical Disorders of the Autonomic Nervous System. Oxford: Oxford University Press. p 312-333.

Ewing DJ. 1993. Noninvasive evaluation of heart rate: The time domain. In: Low PA, editor. Clinical Autonomic Disorders. Boston: Little, Brown & Co. p 297-314.

Ewing DJ, Borsey DQ, Bellavere F, Clarke BF. 1981. Cardiac autonomic neuropathy in diabetes: comparison of measures of R-R interval variation. Diabetologia 21:18-24.

Ewing DJ, Campbell IW, Clarke BF. 1980. The natural history of diabetic autonomic neuropathy. Q J Med 49:95-108.

Ewing DJ, Clarke BF. 1987. Diabetic autonomic neuropathy: a clinical viewpoint. In: Dyck PJ, Thomas PK, Asbury AK, editors. Diabetic neuropathy. Philadelphia: WB Saunders. p 66-88.

Ewing DJ, Martyn CN, Young RJ, Clarke BF. 1985. The value of cardiovascular autonomic function tests: 10 years experience in diabetes. Diabetes Care 8:491-498.

Fernandez O. 2002. Mechanisms and current treatments of urogenital dysfunction in multiple sclerosis. J Neurol 249:1-8.

Flachenecker P, Reiners K, Krauser M, Wolf A, Toyka KV. 2001. Autonomic dysfunction in multiple sclerosis is related to disease activity and progression of disability. Mult Scler 7:327-334.

Flachenecker P, Wolf A, Krauser M, Hartung HP, Reiners K. 1999. Cardiovascular autonomic dysfunction in multiple sclerosis: correlation with orthostatic intolerance. J Neurol 246:578-586.

Fowler CJ, Panicker JN, Drake M, Harris C, Harrison SC, Kirby M, Lucas M, Macleod N, Mangnall J, North A, Porter B, Reid S, Russell N, Watkiss K, Wells M. 2009. A UK consensus on the management of the bladder in multiple sclerosis. Postgrad Med J 85:552-559.

Fritsch JM, Eckberg DL, Graves LD, Wallin BG. 1986. Arterial pressure ramps provoke linear increases of heart period in humans. Am J Physiol 251:R1086-1090.

Frohman EM, Frohman TC, Zee DS, McColl R, Galetta S. 2005. The neuro-ophthalmology of multiple sclerosis. Lancet Neurol 4:111-121.

Goldstein I, Siroky MB, Sax DS, Krane RJ. 1982. Neurourologic abnormalities in multiple sclerosis. J Urol 128:541-545.

Gunal DI, Afsar N, Tanridag T, Aktan S. 2002. Autonomic dysfunction in multiple sclerosis: correlation with disease-related parameters. Eur Neurol 48:1-5.

Haensch CA, Jorg J. 2006. Autonomic dysfunction in multiple sclerosis. J Neurol 253 Suppl 1:I3-9.

Heller PH, Perry F, Jewett DL, Levine JD. 1990. Autonomic components of the human pupillary light reflex. Invest Ophthalmol Vis Sci 31:156-162.

Hilz M, Devinsky O, Szczepanska H, Borod J, Marthol H, Tutaj M. 2006. Right ventromedial prefrontal lesions result in paradoxical cardiovascular activation with emotional stimuli. Brain 129:3343-3355.

Hilz MJ. 2002. Quantitative autonomic functional testing in clinical trials. In: Brown R, Bolton C, Aminoff M, editors. Neuromuscular Function and Disease. Philadelphia: W.B. Saunders Company. p 1899-1929.

Hilz MJ, Dutsch M. 2006. Quantitative studies of autonomic function. Muscle Nerve 33:6-20.

Hilz MJ, Koehn J, Kolodny EH, Brys M, Moeller S, Stemper B. 2011a. Metronomic breathing shows altered parasympathetic baroreflex function in untreated Fabry patients and baroreflex improvement after enzyme replacement therapy. J Hypertens 29:2387-2394.

Hilz MJ, Marthol H, Schwab S, Kolodny EH, Brys M, Stemper B. 2010. Enzyme replacement therapy improves cardiovascular responses to orthostatic challenge in Fabry patients. J Hypertens 28:1438-1448.

Hilz MJ, Moeller S, Akhundova A, Marthol H, Pauli E, De Fina P, Schwab S. 2011b. High NIHSS Values Predict Impairment of Cardiovascular Autonomic Control. Stroke 42:1528-1533.

Hilz MJ, Platsch G, Druschky K, Pauli E, Kuwert T, Stefan H, Neundorfer B, Druschky A. 2003. Outcome of epilepsy surgery correlates with sympathetic modulation and neuroimaging of the heart. J Neurol Sci 216:153-162.

Huitinga I, De Groot CJ, Van der Valk P, Kamphorst W, Tilders FJ, Swaab DF. 2001. Hypothalamic lesions in multiple sclerosis. J Neuropathol Exp Neurol 60:1208-1218.

Hulter BM, Lundberg PO. 1995. Sexual function in women with advanced multiple sclerosis. J Neurol Neurosurg Psychiatry 59:83-86.

Jakobsen J. 1990. Pupillary function in multiple sclerosis. Acta Neurol Scand 82:392-395.

Kale N, Magana S, Agaoglu J, Tanik O. 2009. Assessment of autonomic nervous system dysfunction in multiple sclerosis and association with clinical disability. Neurol Int 1:e5.

Karaszewski JW, Reder AT, Maselli R, Brown M, Arnason BG. 1990. Sympathetic skin responses are decreased and lymphocyte beta-adrenergic receptors are increased in progressive multiple sclerosis. Ann Neurol 27:366-372.

Kirkeby HJ, Poulsen EU, Petersen T, Dorup J. 1988. Erectile dysfunction in multiple sclerosis. Neurology 38:1366-1371.

Koldewijn EL, Hommes OR, Lemmens WA, Debruyne FM, van Kerrebroeck PE. 1995. Relationship between lower urinary tract abnormalities and disease-related parameters in multiple sclerosis. J Urol 154:169-173.

Kragt JJ, Hoogervorst EL, Uitdehaag BM, Polman CH. 2004. Relation between objective and subjective measures of bladder dysfunction in multiple sclerosis. Neurology 63:1716-1718.

Kurtzke JF. 1983. Rating neurologic impairment in multiple sclerosis: an expanded disability status scale (EDSS). Neurology 33:1444-1452.

Lanfranchi PA, Somers VK. 2002. Arterial baroreflex function and cardiovascular variability: interactions and implications. Am J Physiol Regul Integr Comp Physiol 283:R815-826.

Legramante JM, Raimondi G, Massaro M, Cassarino S, Peruzzi G, Iellamo F. 1999. Investigating feed-forward neural regulation of circulation from analysis of spontaneous arterial pressure and heart rate fluctuations. Circulation 99:1760-1766.

Lilius HG, Valtonen EJ, Wikstrom J. 1976. Sexual problems in patients suffering from multiple sclerosis. J Chronic Dis 29:643-647.

Linden D, Diehl RR, Berlit P. 1995. Subclinical autonomic disturbances in multiple sclerosis. J Neurol 242:374-378.

Low PA. 1997. Clinical autonomic disorders : evaluation and management, 2nd ed. Philadelphia: Lippincott-Raven.

Low PA, Hilz MJ. 2008. Diabetic autonomic neuropathy. In: Low PA, Bennaroch EE, editors. Clinical autonomic disorders, 3 ed. Philadelphia: Lippincott Williams & Wilkins. p 423-440.

Lowenstein O, Loewenfeld I. 1950. Mutual role of sympathetic and parasympathetic in shaping of the pupillary reflex to light. Arch Neurol Psychiatry 64:341-377.

Lundberg PO. 1981. Sexual dysfunction in female patients with multiple sclerosis. Int Rehabil Med 3:32-34.

Marthol H, Brown CM, Zikeli U, Ziegler D, Dimitrov N, Baltadzhieva R, Hilz MJ. 2006. Altered cerebral regulation in type 2 diabetic patients with cardiac autonomic neuropathy. Diabetologia 49:2481-2487.

Marthol H, Zikeli U, Brown CM, Tutaj M, Hilz MJ. 2007. Cardiovascular and cerebrovascular responses to lower body negative pressure in type 2 diabetic patients. J Neurol Sci 252:99-105.

Mathias C, Bannister R. 1993. Investigations of autonomic disorders. In: R B, editor. Autonomic Failure. Oxford: Oxford medical publications. p 255-290.

Merkelbach S, Haensch CA, Hemmer B, Koehler J, Konig NH, Ziemssen T. 2006. Multiple sclerosis and the autonomic nervous system. J Neurol 253 Suppl 1:I21-25.

Miller H, Simpson CA, Yeates WK. 1965. Bladder Dysfunction in Multiple Sclerosis. Br Med J 1:1265-1269.

Mutani R, Clemente S, Lamberti A, Monaco F. 1982. Assessment of autonomic disturbances in multiple sclerosis by measurement of heart rate responses to deep breathing and to standing. Ital J Neurol Sci 3:111-114.

Nakipoglu GF, Kaya AZ, Orhan G, Tezen O, Tunc H, Ozgirgin N, Ak F. 2009. Urinary dysfunction in multiple sclerosis. J Clin Neurosci 16:1321-1324.

Nasseri K, TenVoorde BJ, Ader HJ, Uitdehaag BM, Polman CH. 1998. Longitudinal follow-up of cardiovascular reflex tests in multiple sclerosis. J Neurol Sci 155:50-54.

Natale E, Marzi CA, Bricolo E, Johannsen L, Karnath HO. 2007. Abnormally speeded saccades to ipsilesional targets in patients with spatial neglect. Neuropsychologia 45:263-272.

Nordenbo AM, Boesen F, Andersen EB. 1989. Cardiovascular autonomic function in multiple sclerosis. J Auton Nerv Syst 26:77-84.

Oppenheimer DR. 1976. Demyelinating disease. In: Blackwood W, Corsellis JAN, editors. Greenfield`s Neuropathology. London: Edward Arnold. p 470-499.

Ormezzano O, Cracowski JL, Quesada JL, Pierre H, Mallion JM, Baguet JP. 2008. EVAluation of the prognostic value of BARoreflex sensitivity in hypertensive patients: the EVABAR study. J Hypertens 26:1373-1378.

Parati G, Di Rienzo M, Bertinieri G, Pomidossi G, Casadei R, Groppelli A, Pedotti A, Zanchetti A, Mancia G. 1988. Evaluation of the baroreceptor-heart rate reflex by 24-hour intra- arterial blood pressure monitoring in humans. Hypertension 12:214-222.

Parati G, Di Rienzo M, Mancia G. 2001. Dynamic modulation of baroreflex sensitivity in health and disease. Ann N Y Acad Sci 940:469-487.

Patel HD, Livsey SA, Swann RA, Bukhari SS. 2005. Can urine dipstick testing for urinary tract infection at point of care reduce laboratory workload? J Clin Pathol 58:951-954.

Pentland B, Ewing DJ. 1987. Cardiovascular reflexes in multiple sclerosis. Eur Neurol 26:46-50.

Piha SJ, Halonen JP. 1994. Infrared pupillometry in the assessment of autonomic function. Diabetes Res Clin Pract 26:61-66.

Pitzalis MV, Mastropasqua F, Passantino A, Massari F, Ligurgo L, Forleo C, Balducci C, Lombardi F, Rizzon P. 1998. Comparison between noninvasive indices of baroreceptor sensitivity and the phenylephrine method in post-myocardial infarction patients. Circulation 97:1362-1367.

Reulen JP, Sanders EA, Hogenhuis LA. 1983. Eye movement disorders in multiple sclerosis and optic neuritis. Brain 106 (Pt 1):121-140.

Robbe HW, Mulder LJ, Ruddel H, Langewitz WA, Veldman JB, Mulder G. 1987. Assessment of baroreceptor reflex sensitivity by means of spectral analysis. Hypertension 10:538-543.

Rougier MB, Tilikete C. 2008. [Ocular motor disorders in multiple sclerosis]. J Fr Ophtalmol 31:717-721.

Rudiger H, Klinghammer L, Scheuch K. 1999. The trigonometric regressive spectral analysis- a method for mapping of beat-to-beat recorded cardiovascular parameters on to frequency domain compared with Fourier transformation. Comput Methods Programs Biomed 58:1-15.

Saul JP, Berger RD, Chen MH, Cohen RJ. 1989. Transfer function analysis of autonomic regulation. II. Respiratory sinus arrhythmia. Am J Physiol 256:153-161.

Senaratne MP, Carroll D, Warren KG, Kappagoda T. 1984. Evidence for cardiovascular autonomic nerve dysfunction in multiple sclerosis. J Neurol Neurosurg Psychiatry 47:947-952.

Serra A, Derwenskus J, Downey DL, Leigh RJ. 2003. Role of eye movement examination and subjective visual vertical in clinical evaluation of multiple sclerosis. J Neurol 250:569-575.

Smith S. 1993. Pupil function: Tests and disorders. In: Bannister R, Mathias CJ, editors. Autonomic Failure: a textbook of clinical disorders of the autonomic nervous system, 3 ed. Oxford: Oxford Medical Publications. p 421-441.

Smith SA, Dewhirst RR. 1986. A simple diagnostic test for pupillary abnormality in diabetic autonomic neuropathy. Diabet Med 3:38-41.

Sterman AB, Coyle PK, Panasci DJ, Grimson R. 1985. Disseminated abnormalities of cardiovascular autonomic functions in multiple sclerosis. Neurology 35:1665-1668.

Stewart JD, Fowler CJ. 1999. Neurology of bladder, bowel and sexual dysfunction. J Clin Neuromuscul Dis 1:112.

Stuve O, Oksenberg J. 1993. Multiple Sclerosis Overview.

Suarez GA, Opfer-Gehrking TL, Offord KP, Atkinson EJ, O'Brien PC, Low PA. 1999. The Autonomic Symptom Profile: a new instrument to assess autonomic symptoms. Neurology 52:523-528.

Sundkvist G, Almer LO, Lilja B. 1979. Respiratory influence on heart rate in diabetes mellitus. Br Med J 7:924-925.

Sykora M, Diedler J, Rupp A, Turcani P, Steiner T. 2009. Impaired baroreceptor reflex sensitivity in acute stroke is associated with insular involvement, but not with carotid atherosclerosis. Stroke 40:737-742.

Vita G, Fazio MC, Milone S, Blandino A, Salvi L, Messina C. 1993. Cardiovascular autonomic dysfunction in multiple sclerosis is likely related to brainstem lesions. J Neurol Sci 120:82-86.

Wheeler T, Watkins PJ. 1973. Cardiac denervation in diabetes. Br Med J 4:584-586.

Zivadinov R, Zorzon M, Bosco A, Bragadin LM, Moretti R, Bonfigli L, Iona LG, Cazzato G. 1999. Sexual dysfunction in multiple sclerosis: II. Correlation analysis. Mult Scler 5:428-431.

Zorzon M, Zivadinov R, Bosco A, Bragadin LM, Moretti R, Bonfigli L, Morassi P, Iona LG, Cazzato G. 1999. Sexual dysfunction in multiple sclerosis: a case-control study. I. Frequency and comparison of groups. Mult Scler 5:418-427.

IX) Procedures suited for non-invasively assessing correlations between clinical MS severity and autonomic nervous system dysfunction in a cross-sectional and prospective 36 months follow-up study of patients with preselected disease severity and treatment history

Procedure	Assessment	Rationale
Expanded Disability Status Scale (EDSS)	Assessment of the degree of neurological impairment	Impairment of neurological function is common in MS.<br>Standardized tests evaluating neurological function are suited and commonly used to monitor the degree and progression of neurological impairment. EDDSS and MSFC serve as clinical parameters for cross-sectional and prospective testing of correlations between parameters of autonomic dysfunction and clinical MS severity
Multiple Sclerosis Functional Composite (MSFC)	Assessment of lower extremity, upper extremity and cognitive function	
Infrared light reflex pupillography	Assessment of afferent, central, and efferent pathways involved in the visual process and the adjustment of the pupil	Frequent involvement of brain areas contributing to pupillary light reflex responses in MS pathology can be easily assessed by infrared light reflex pupillography.<br>Parameters of autonomic pupillary dysfunction serve as indices of autonomic dysfunction due to MS pathology and progression in a cross-sectional and prospective comparison of clinical and autonomic dysfunction
Video-Nystagmography	Testing of horizontal saccadic eye movements	Impairment of saccadic eye movements may be predictive of MS severity and progression.<br>Video-nystagmographic assessment of saccadic eye movements may be suited for cross-sectional and prospective correlations with scores of clinical MS severity.
Autonomic challenge maneuvers	Heart rate and blood pressure responses and spectral powers of autonomic modulation during active standing	Alteration of the cardiovascular control in MS patients may result in orthostatic intolerance reflecting altered sympathetic and parasympathetic outflow to the cardiovascular system.<br>Standing-up assesses changes in baroreflex function, a parameter reflecting disease prognosis and therefore suited for a cross-sectional and prospective evaluation of correlations between clinical MS severity and autonomic dysfunction.
Autonomic challenge maneuvers	Blood pressure and heart rate responses during the Valsalva maneuver	Baroreflex mediated reflex bradycardia after release of the expiratory strain during the VM is modulated by central areas of autonomic control and may be impaired in MS patients.<br>Baroreflex sensitivity is non-invasively assessed by the Valsalva maneuver and serves as additional parameter reflecting disease prognosis and is therefore suited for a cross-sectional and prospective evaluation of correlations between clinical MS severity and autonomic dysfunction.
Autonomic challenge maneuvers	Heart rate variation during 3-minute deep breathing	MS patients may have abnormal heart rate modulation with metronomic deep breathing, as the respiratory modulation of

		heart rate involves multiple central and peripheral structures of the autonomic nervous system. Metronomic deep breathing assesses the ability to augment parasympathetic cardiac modulation non-invasively and is therefore suited for a cross-sectional and prospective evaluation of correlations between clinical MS severity and autonomic dysfunction.
Autonomic challenge maneuvers	Blood pressure response to sustained handgrip test	Tests assessing sympathetic function may reveal abnormalities in cardiovascular control in MS patients.<br>Blood pressure testing during the sustained handgrip test non-invasively determines the ability to enhance sympathetic vasomotor responses, and is therefore suited for a cross-sectional and prospective evaluation of correlations between clinical MS severity and autonomic dysfunction.
dip stick	Assessment of the urine status and signs of urinary tract infection	Bladder infections are a common cause for deterioration in clinical status and increased mortality in MS and may be secondary to autonomic bladder dysfunction.<br>Dip stick urine analysis non-invasively determines urinary tact infection and is therefore suited for a cross-sectional and prospective evaluation of correlations between autonomic bladder dysfunction and clinical MS severity.
Post-voiding residual urine sonography	Assessment of bladder function	Dysfunction of bladder emptying is a frequent and common cause for secondary complications in MS.<br>Post-voiding residual urine sonography non-invasively determines intra-vesical residual urine volume, and is therefore suited for a cross-sectional and prospective evaluation of correlations between autonomic bladder dysfunction and clinical MS severity.
Uroflowmetry	Assessment of bladder function	Dysfunction of bladder emptying is a frequent and common cause for secondary complications in MS.<br>Uroflowmetry non-invasively assesses bladder voiding largely controlled by the autonomic nervous system, and is therefore suited for a cross-sectional and prospective evaluation of correlations between autonomic bladder dysfunction and clinical MS severity.
Questionnaires	Assessment of sexual dysfunction, bladder dysfunction and autonomic symptoms	Impairment of sexual, bladder and general autonomic function is frequent in MS.<br>Questionnaires non-invasively evaluate prevalence and severity of sexual, bladder and general autonomic dysfunction, and are therefore suited for a cross-sectional and prospective evaluation of correlations between sexual, bladder and general autonomic dysfunction and clinical MS severity.

X) Timeline of Assessment

Baseline after patient fulfilling enrollment criteria has agreed to participate in study and has signed informed consent, but before change in disease modifying treatment	Within 24 hours after change in disease modifying treatment	After 6 months ± 2 weeks upon enrollment	After 12 months ± 2 weeks upon enrollment	After 24 months ± 2 weeks upon enrollment	After 36 months ± 2 weeks upon enrollment
