# Supplementary material for: Central Autonomic Dysfunction Delays Recovery of Fingolimod Induced Heart Rate Slowing
Source: PLoS One. 2015 Jul 6;10(7):e0132139. doi: 10.1371/journal.pone.0132139 (PMC4493029; doi:10.1371/journal.pone.0132139)
Supplement: S1 TREND Checklist — (DOC) [file pone.0132139.s002.doc]

# Research checklist according to TREND Statement checklist

| **Paper Section/Topic** | **Item**  **No** |  | **On page #** |
| --- | --- | --- | --- |
| **TITLE and ABSTRACT** | 1 | Central autonomic dysfunction delays recovery of Fingolimod induced heart rate slowing | 1-2 |
| **INTRODUCTION** |  |  |  |
| *Background* | 2 | It is unknown why some MS patients have prolonged or more severe HR-slowing, or even develop bradycardia or atrioventricular blocks upon first dose of Fingolimod. It seems unlikely that this risk is primarily due to cardiac abnormalities such as individual variations in the sensitivity of myocardial potassium channels towards Fingolimod. Instantaneous HR-adjustment and counter-regulation depend on the integrity of the central autonomic network (CAN). MS-lesions frequently afflict CAN structures and may thus cause autonomic dysfunction including cardiovascular and baroreflex dysregulation. | 3-4 |
| **METHODS** |  |  |  |
| *Participants* | 3 | Participant recruitment: Recruitment was based on presenting symptoms, and the fact that participants were appointed to receive Fingolimod treatment. |  |
| *Interventions* | 4 | All patients received their first dosage of Fingolimod within the hour after we had ended the autonomic tests. Participants were tested between 9 AM and 2 PM, after a resting period of at least 40 min that ensured a stable cardiovascular situation. None of our patients had any electrocardiogram (ECG) abnormalities prior to Fingolimod-initiation. | 5 |
| *Objectives* | 5 | We hypothesize that MS-related CAN dysfunction impairs counter-regulation of the common though clinically mostly harmless Fingolimod-induced HR-slowing and thus delays recovery of HR-slowing beyond six hours or, in rare cases; even causes bradycardia or atrioventricular blocks.  We therefore evaluated whether standard autonomic cardiovascular testing reveals autonomic cardiovascular dysfunction prior to Fingolimod-initiation in those MS patients who need more than six hours after Fingolimod-initiation to re-increase their HR. | 4 |
| *Outcomes* | 8 | To assess cardiovascular autonomic modulation, we recorded HR as RR-intervals (RRI; [ms]) by 3-lead electrocardiography, beat-to-beat systolic and diastolic blood-pressure (BPsys, BPdia; [mmHg]) by finger-pulse photoplethysmography (Portapress; TPD Biomedical Instrumentation, Amsterdam, The Netherlands), and respiratory frequency (RESP [min-1]) by chest impedance measurements during three minutes at supine rest, and during autonomic challenge by active standing-up, metronomic deep breathing (MDB), during three Valsalva maneuvers (VM), and a three minute sustained-handgrip exercise.  To estimate MS severity, we used the Expanded Disability Status Scale (EDSS) and the Multiple Sclerosis Functional Composite (MSFC). | 5-6 |
| *Sample size* | 7 | Sample size calculation was not done. |  |
| *Assignment Method* | 8 | Individual patients were assigned to Fingolimod based on presenting symptoms. All patients were appointed to receive Fingolimod treatment.  Patients who had received previous disease modifying treatments (DMT) were taken off their previous medication for at least the period consistent with current recommendation, i.e. for at least 6 months if the patients were on cytotoxic drugs (e.g. mitoxantrone), for at least 2-3 months if the patients were on natalizumab. Fingolimod could generally be started immediately after discontinuation of an interferon or glatiramer acetate.  Participants with other diseases than MS or on medication affecting the autonomic nervous system were excluded from the study.  We compared results to those of 20 age-matched healthy volunteers. | 5 |
| *Blinding* | 9 | The staff member performing the assessments was not involved in implementing any aspect of the intervention. |  |
| *Unit of analysis* | 10 | As recommended by the European Medicines Agency, we monitored HR in the MS patients after Fingolimod-initiation for at least six hours or longer if HR was at the lowest value 6 hours after the first dose (<http://www.ema.europa.eu/ema>).  Upon Fingolimod-initiation, 7 of 21 patients had prolonged HR-slowing.  We compared results of patients with and without prolonged (> six hours) HR-slowing after Fingolimod-initiation and of healthy persons. | 5 |
| *Statistical methods* | 12 | Data were tested for normal distribution using the Shapiro-Wilk test. We assessed differences in bio-signals and autonomic parameters at rest and during autonomic challenge between patients without and with prolonged HR-slowing (beyond 6 hours) upon Fingolimod-initiation and controls by analysis of variance for repeated measurements (ANOVA, general linear model). We used ‘challenge’ (rest, active standing, MDB, VM, and SHGE) as within-subject factors and ‘group’ (patients with and without prolonged HR-slowing upon Fingolimod-initiation, and controls) as between-subject factor. Suitability of the ANOVA model was assessed by Mauchly’s test of sphericity. In case of violation of the sphericity assumption, the Greenhouse Geisser correction was employed. In case of significant ANOVA results, we performed post-hoc single comparisons. For comparison of EDSS-, MSFC-scores, and the lowest HR-values upon Fingolimod-initiation between patients with and without prolonged HR-slowing, we used the Mann–Whitney U-test. We used the Chi-square test to assess gender differences between groups. Significance was set at p<0.05. For data analysis, we used a commercially available statistical program (IBM SPSS Statistics for Windows, Version 20.0. Armonk, NY, USA). | 9 |
| **RESULTS** |  |  |  |
| *Participant flow* | 12 | See fig. 1 | Fig. 1 |
| *Recruitment* | 13 | The recruitment period was between November 2012 and July 2013. | 5 |
| *Baseline Data* | 14 | 21 patients with relapsing-remitting MS were monitored immediately before Fingolimod-initiation.  Upon Fingolimod-initiation, 7 of 21 patients had prolonged HR-slowing. Patients without prolonged HR-slowing: Number of patients 14; Sex [female/male] 9/14; Age [year] 34.2±6.5; Weight [kg] 71.2±18.2; Height [cm] 169.6±7.6; EDSS 2.0 (1.5-2.5); MSFC 0.07 (-0.41-0.40); Number of relapses in previous year 1 (1-2); Interval in years from symptom-onset to date of examination 5.9 (3.5-11.2).  Patients with prolonged HR-slowing: Number of patients 7; Sex [female/male] 3/7; Age [year] 34.9±7.2; Weight [kg] 74.2±17.5; Height [cm] 180.3±12.4; EDSS 2.0 (1.5-3.0); MSFC 0.41 (-0.17-0.61); Number of relapses in previous year 1 (1-2); Interval in years from symptom-onset to date of examination 6.4 (3.0-14.1).  Healthy persons: Number of patients 20; Sex [female/male] 10/10; Age [year] 30.1±12.5; Weight [kg] 72.5±3.5; Height [cm] 179.5±12.0. | 11, Table 1 |
| *Baseline equivalence* | 15 | The patient and control groups did not statistically differ with respect to demographic data (gender, age, weight and height; p > 0.05) | Table 1 |
| *Numbers analyzed* | 16 | Each analysis involved all patients and control participants |  |
| *Outcomes and estimation* | 17 | See result section |  |
| *Ancillary analyses* | 18 | We did not perform subgroup analysis. |  |
| *Adverse events* | 19 | The methods of autonomic challenge maneuvers did not induce adverse events.  None of the patients developed any ECG changes during or at the end of the first Fingolimod dosing, and all patients remained clinically asymptomatic. In 14 patients, HR re-increased within 6 hours, while seven patients had HR-slowing for more than 6 hours, and only re-increased HR within 8 hours. | 11 |
| **DISCUSSION** |  |  |  |
| *Interpretation* | 20 | Our autonomic findings show that the seven patients who had a delayed HR re-increase after Fingolimod-initiation have a subtle, clinically not overt central autonomic dysfunction. Most likely, MS-related CAN lesions compromise autonomic adjustment to cardiovascular challenge and thus delay counter-regulation of HR-slowing upon Fingolimod-initiation. | 17 |
| *Generalizability* | 21 | We speculate that more pronounced dysfunction of central autonomic control might also contribute to the pathophysiology of the rare cases of clinically relevant cardiac arrhythmias upon Fingolimod-initiation. | 17 |
| *Overall Evidence* | 22 | See discussion section | 14-17 |
